# *Grtp1* Safeguards Against Paternal Reproductive Aging and Intergenerational Behavioral Deficits by Preserving Redox Homeostasis

**DOI:** 10.1002/advs.77463

**Published:** 2026-09-27

**Authors:** Yingdong Liu, Shanyao Liu, Haixin Liang, Jiani Yang, Yanhe Li, Jiqing Yin, Xueguang Zhang, Yan Zhang, Dandan Bai, Zihui Yan, Zhuoao Zhang, Yanping Jia, Kuisheng Liu, Yifan Sheng, Jiani Xiang, Chenxiang Xi, Baoxing Dong, Xinyi Lei, Jiayu Chen, Hong Wang, Yi Guo, Yanping Zhang, Shaorong Gao, Wenqiang Liu

**Affiliations:** ^1^ Shanghai Key Laboratory of Maternal Fetal Medicine Clinical and Translational Research Center of Shanghai First Maternity and Infant Hospital Shanghai Institute of Maternal‐Fetal Medicine and Gynecologic Oncology Shanghai Key Laboratory of Signaling and Disease Research Frontier Science Center for Stem Cell Research School of Life Sciences and Technology Tongji University Shanghai China

**Keywords:** behavioral abnormalities, DNA methylation, growth hormone, *Grtp1*, male reproductive aging, redox homeostasis

## Abstract

Oxidative stress is a primary mediator of male reproductive decline and offspring behavioral abnormalities. Unlike females, males exhibit enhanced antioxidant capacity and sustained fertility, but the mechanism is unclear. Here, using single‐cell transcriptomics and cross‐sex comparisons, we identify *Grtp1* as critical for counteracting male reproductive aging and protecting offspring from behavioral deficits by preserving germline redox homeostasis and sperm DNA methylation stability. *Grtp1* ablation recapitulates aging phenotypes, including oxidative stress, mitochondrial dysfunction, impaired spermatogenesis, and offspring anxiety and social deficits. Mechanistically, GRTP1 interacts with PRDX1/4 and TXN to maintain germline redox balance. Notably, growth hormone treatment in aged males rescues germline redox homeostasis, restores abnormal sperm DNA methylation and quality, and ameliorates offspring behavioral deficits in a *Grtp1*‐dependent manner. Our study establishes GRTP1 as a key redox safeguard against male germline aging and highlights the GH‐GRTP1 axis as a promising therapeutic target for age‐related reproductive and intergenerational behavioral deficit.

## Graphical abstract

This study identifies that *Grtp1* maintains germline redox homeostasis by interacting with PRDX1/4‐TXN to antagonize paternal reproductive aging. Growth hormone restores *Grtp1* expression, rectifies aberrant sperm DNA methylation, and ameliorates intergenerational anxiety and social deficits, highlighting the GH‐GRTP1 axis as a promising intervention target.

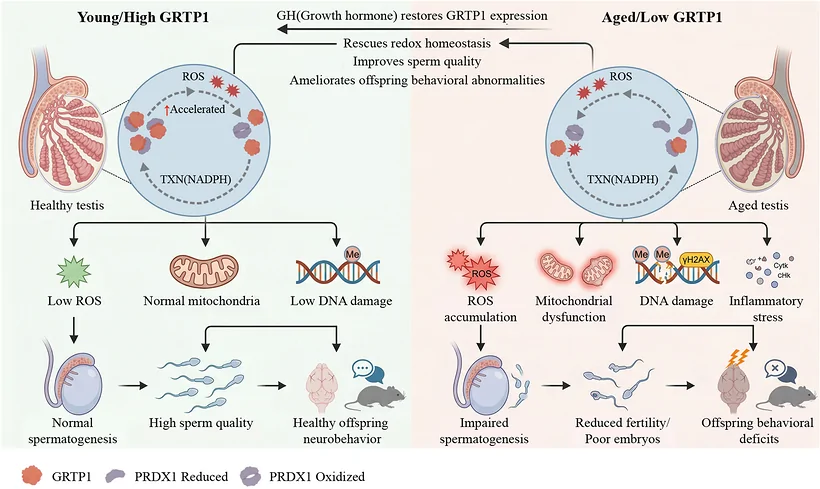

## 1 Introduction

Oxidative stress is a central driver of age‐related reproductive dysfunction, with profound implications for both parental fertility and offspring health. Unlike females, males exhibit a considerably extended reproductive lifespan, may be attributed to the peroxiredoxins (PRDX1/4), thioredoxin (TXN), and glutathione (GSH) in the testis endow it with stronger antioxidant stress capacity [1, 2]. Yet, male fertility inevitably declines with advancing age [3, 4], and advanced paternal age (APA) is increasingly linked to progressive fertility impairment, characterized by quantitative and qualitative changes in spermatogenesis [5]. At the cellular level, aging leads to a gradual depletion of spermatogonial stem cells, impairing the maintenance of the spermatogenic lineage and altering germ cell populations [6, 7]. Concurrently, germ cells accumulate age‐related DNA damage, reflected in an elevated sperm DNA fragmentation index (DFI), which is clinically linked to higher risks of pregnancy loss and potential neurodevelopmental issues in offspring [8]. These changes occur within a deteriorating testicular microenvironment, marked by age‐related hormonal dysregulation, seminiferous tubule atrophy, and disrupted somatic‐germ cell communication [9]. Somatic components, such as leydig and peritubular myoid cells, further contribute through chronic inflammation and dysregulated DNA repair, potentially exacerbated by oxidative stress [10, 11, 12]. Despite the established role of redox imbalance in male reproductive aging, the molecular mechanisms that sustain germline redox homeostasis during aging and protect against intergenerational phenotypic transmission remain poorly defined.

Notably, the antioxidant defense system centered on peroxiredoxins (PRDX1/4) and thioredoxin (TXN) in the testis plays a central regulatory role in maintaining redox homeostasis in germ cells [13, 14]. By efficiently scavenging reactive oxygen species (ROS), this system directly safeguards genomic stability and the fidelity of epigenetic reprogramming in spermatogonial stem cells and developing spermatids, thereby constituting a critical molecular barrier against age‐related reproductive decline [15]. However, the efficacy of this critical defense system declines with advancing age, leading to the accumulation of oxidative damage and increased genomic instability [16]. These fundamental impairments within germ cells not only directly compromise male fertility but may also transmit aberrant genetic and epigenetic information to offspring via sperm [17, 18, 19, 20, 21]. Epidemiological evidence confirms a significantly elevated risk of neurodevelopmental disorders, including autism spectrum disorder, among offspring of older fathers [22]. Offspring of fathers over 45 face an approximately 75% increased risk of autism spectrum disorder compared to those with fathers aged 20–24 [23]. Beyond genetic mutations, altered sperm DNA methylation patterns and changes in non‐coding RNAs, such as miRNAs and tsRNAs, have been implicated in neurological disorders in the next generation [24, 25, 26, 27]. In summary, advanced paternal age elevates offspring risk through an underlying biological pathway that involves oxidative decline, genetic instability, and epigenetic alterations in germ cells.

This study integrated single‐cell and bulk RNA sequencing data from testes of young and aged male mice, along with transcriptomic data from ovaries of young female mice as controls, to conduct a cross‐sex comparative analysis. This approach identified *Grtp1* as a potential core regulatory factor in aged male testes. Mechanistic investigations demonstrated that the GRTP1 protein forms a functional complex with key components of the intracellular antioxidant system, and loss of this interaction leads to oxidative stress accumulation and compromised genomic stability in spermatogonial cells. Notably, *Grtp1* deficiency not only mimics the phenotypic characteristics of naturally aged testes but also induces anxiety‐like and abnormal social behaviors in offspring, replicating an intergenerational effect similar to that of advanced paternal age. Such intergenerational behavioral deficits may be transmitted via altered sperm DNA methylation, a well‐recognized epigenetic mechanism underlying paternal age‐related inheritance. Importantly, growth hormone intervention was shown to achieve multi‐level restorative effects, from improved testicular function to corrected offspring phenotypes, by specifically upregulating *Grtp1* expression. These findings provide new insights into the mechanisms of testicular aging and open new avenues for intervening in age‐related reproductive decline by targeting the *Grtp1* pathway.

## 2 Results

### 2.1 *Grtp1* is a Key Downregulated Factor in Male Reproductive Aging

**FIGURE 1:**
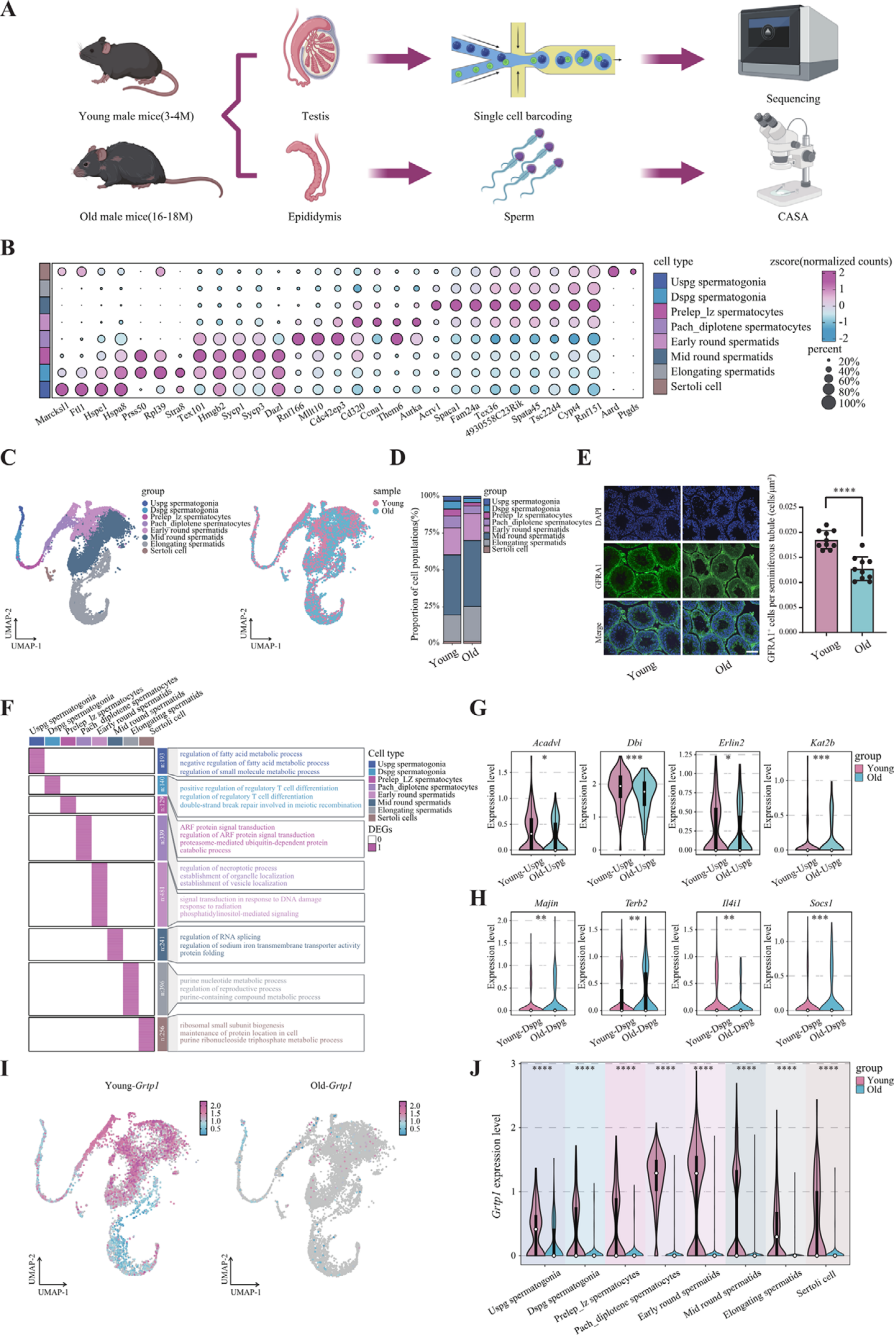
Identification of *Grtp1* as a key downregulated factor in aged testes via integrated multi‐omics analysis. (A) Schematic overview of the experimental strategy to assess the impact of aging on male reproduction, integrating morphological, transcriptomic, and functional analyses. (B) Heatmap of representative marker‑gene expression used for annotating testicular cell types. (C) UMAP visualization of single‐cell RNA sequencing data from young and aged testes, colored by major cell types. (D) Bar plot showing the proportional abundance of testicular cell types in young vs. aged groups. (E) Immunofluorescence validation of the age‐related decline in undifferentiated spermatogonia using the specific marker GFRA1 (scale = 100µm). Quantification confirms a significant decrease in GFRA1‑positive cells in aged male testes (n = 10), consistent with the scRNA‑seq results in Figure 1D. (F) Binary DEG mapping and functional enrichment. (Left) Heatmap indicating the presence (1, purple) or absence (0, white) of top DEGs across major testicular cell types between young and aged mice. (Right) Gene Ontology (GO) analysis of biological processes associated with cell‑type‑specific DEGs. (G, H) Violin plots of representative DEGs in undifferentiated and differentiated spermatogonia. Upper panel: DEGs linked to metabolic dysregulation (e.g., *Acadvl*, *Dbi*) (G). Lower panel: DEGs associated with DNA‑damage‑related processes (e.g., *Majin*, *Terb2*) (H). (I). Feature plots depicting *Grtp1* expression in testicular tissues from young and aged males (single‑cell transcriptomics). (J) Violin plot showing *Grtp1* expression levels across testicular cell clusters. Data in Figure 1E, presented as mean ± s.d. (n = 10 independent experiments), were analyzed using a two‐tailed Student's unpaired t‐test, whereas data in Figure 1G,H,J were analyzed with the Wilcoxon rank‐sum test. Statistical significance is indicated as ^*^ *P* < 0.05, ^**^ *P* < 0.01, ^***^ *P* < 0.001, and ^****^ *P* < 0.0001.

To investigate the effects of aging on male reproduction, we systematically evaluated testicular morphological alterations, transcriptomic changes, and functional sperm parameters (Figure 1A). Hematoxylin and eosin (H&E) staining revealed prominent degenerative alterations in the aged testes, including seminiferous tubule atrophy and thinning of the seminiferous epithelium (Figure S1A). Given the inherent high cellular heterogeneity of testicular tissue, we employed 10X Genomics single‐cell RNA sequencing to precisely characterize molecular changes across distinct cell populations during aging. Following rigorous quality control, a total of 16 167 high‐quality cells were retained for downstream analysis (7338 from the young group and 8829 from the old group). Uniform manifold approximation and projection (UMAP) clustering identified seven spermatogenic cell types and sertoli cells, including undifferentiated spermatogonia cells (Uspg, *Ftl1*); differentiated spermatogonia cells (Dspg, *Stra8*); prelep_lz spermatocytes (preleptotene, leptotene, and zygotene spermatocytes, *Sycp1*); pach_diplotene spermatocytes (pachytene and diplotene spermatocytes, *Mllt10*); early round spermatids (*Ccna1*); mid round spermatids (*Acrv1*); elongating spermatids (*Rnf151*) and sertoli cells (*Aard*) (Figure 1B,C and Figure S1C).

Single‐cell profiling confirmed a significant reduction in the proportion of undifferentiated spermatogonia (Uspg) in aged testes, which was further validated by GFRA1 immunolabeling of this cell population (Figure 1D,E). To characterize age‐related transcriptional changes in each testicular cell populations, we identified specific differentially expressed genes (DEGs) and performed Gene Ontology (GO) functional analyses (Figure 1F and Figure S1D). Notably, single‐cell transcriptomic analysis revealed that aging induces significant transcriptional dysregulation as early as the initial stage of spermatogenesis (Uspg and Dspg stages)‐predominantly characterized by exacerbated inflammatory responses and disrupted energy metabolism (Figure 1G,H). These early molecular defects progressively amplify throughout spermatogenic progression, leading to cascading gene expression abnormalities in subsequent spermatocytes and spermatids, including significant dysregulation of meiotic regulators and spermiogenesis‐related genes.

Consistent with single‐cell data, bulk mRNA‐seq analysis of testicular tissues demonstrated significant differential gene expression in aged tissues enriched in immune‐inflammatory responses and related aging processes (Figure S1E,F). The cytokine array confirmed significant upregulation of pro‐inflammatory factors MIP‐1g, IL‐1a, and ICAM‐1 in aged testicular tissues (Figure S1B). These results demonstrate that aged testes undergo extensive alterations at both the single‐cell and tissue levels.

Given that the male reproductive system exhibits stronger anti‐aging capabilities compared to the female, we hypothesized it expresses male‐specific protective factors sustaining reproductive function. We compared testicular transcriptomes between young and aged males, integrating young female ovarian data for cross‐sex comparison to identify testis‐enriched, age‐regulated candidates. We first intersected testis‐highly expressed genes (vs. ovaries) with age‐downregulated testicular genes (young vs. aged), yielding seven candidates (Figure S2A,B). Among these, *Grtp1* showed robust expression in young testes, drastically reduced to near‐undetectable levels in aged testes, and scRNA‐seq confirmed it as a top‐ranked DEG across all testicular cell populations (Figure S2C). UMAP visualization consistently revealed significantly downregulated *Grtp1* expression across all cell clusters in aged groups (Figure 1I,J). These multiple lines of evidence suggest that *Grtp1* may play a critical role in regulating male reproductive aging.

### 2.2 *Grtp1* Depletion in Young Males Recapitulates Testicular Aging Phenotypes

**FIGURE 2:**
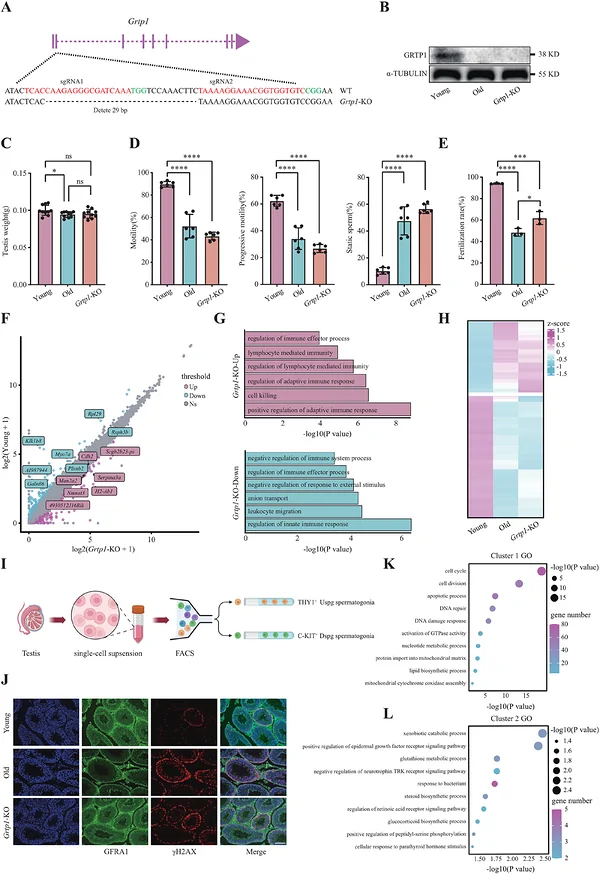
*Grtp1* deficiency impairs sperm function and recapitulates the inflammatory transcriptomic landscape of testicular aging. (A) Schematic of the CRISPR‐Cas9 strategy used to generate *Grtp1*‑knockout (*Grtp1*‐KO) mice, showing a 29‑bp deletion in exon 2. (B) Western blot confirming the loss of GRTP1 protein in testicular tissues from *Grtp1*‑KO mice compared with young wild‑type controls. (C) Testicular weight in young, aged, and *Grtp1*‑KO mice. (D) Sperm motility parameters (total motility, progressive motility, and static fraction) in young, aged, and *Grtp1*‐KO groups. (E) Bar graph depicting the comparison of fertilization rates across young wild‐type, aged wild‐type, and *Grtp1*‐KO mouse groups. (F) Heatmap of differentially expressed genes (DEGs) between young wild‑type and young *Grtp1*‑KO testicular tissues. (G) Gene Ontology (GO) enrichment analysis of up‑ and down‑regulated DEGs in young *Grtp1*‑KO vs. wild‑type testes. (H) Heatmap displaying the expression patterns of DEGs, highlighting the convergent transcriptomic landscape in aged and *Grtp1*‐KO testes compared to the young group. (I) Flow chart depicting the process of isolating undifferentiated (THY1^+^) and differentiated (C‐KIT^+^) spermatogonial populations from enzymatically digested testicular tissue. (J) Representative immunofluorescence images of γ‑H2AX foci (red) in testicular sections from young, aged, and *Grtp1*‑KO mice. Sections were co‑stained for the undifferentiated spermatogonia marker GFRA1 (green) and counterstained with DAPI (blue) (scale = 100µm). (K, L) GO terms enriched for genes that were consistently upregulated (K, Cluster 1) or downregulated (L, Cluster 2) in both aged and *Grtp1*‐KO testes relative to young controls. Data in Figure 2C–E are presented as mean ± s.d. (C, n = 10; D, n = 6; E, n = 3 independent experiments). Statistical significance was determined by two‐tailed Student's unpaired t‐tests. Statistical significance is indicated as ns, not significant; ^*^ *P* < 0.05; ^****^ *P* < 0.0001.

Given the testis‐enriched expression and age‐dependent downregulation of *Grtp1*, we utilized CRISPR‐Cas9 technology to generate *Grtp1*‐knockout (*Grtp1*‐KO) mouse to dissect its functional role (Figure 2A) [28]. Sanger sequencing confirmed a 29‐bp deletion in exon 2 of the *Grtp1* gene (Figure S3A), and Western blot verified the complete absence of GRTP1 protein in *Grtp1*‐KO mice (Figure 2B).

We compared *Grtp1*‐KO (3‐4 M) males with young (3–4 M) and aged (16–18 M) controls to evaluate reproductive phenotypes. No significant differences in testicular weight were observed among the three groups (Figure 2C). Computer‐assisted sperm analysis (CASA) results demonstrated severe sperm motility defects in both aged and *Grtp1*‐KO groups: the percentage of progressive motile sperm significantly decreased from 62% in the young group to 34% in the aged group and 27% in the *Grtp1*‐KO group (Figure 2D). In vitro fertilization (IVF) experiments confirmed impaired fertilization capacity. The fertilization rate in the young control group reached 94%, while the aged and *Grtp1*‐KO groups exhibited significantly reduced rates of 49% and 62%, respectively (Figure 2E). Moreover, embryos derived from these two groups displayed significant developmental defects during pre‐implantation stages. The blastocyst formation rate decreased from 80% in the young group to 50% in the aged group and 54% in the *Grtp1*‐KO group, with no statistically significant difference observed between the aged and *Grtp1*‐KO groups (Figure S3B,C).

To elucidate the molecular mechanisms, we performed testicular bulk RNA‐seq. *Grtp1*‐KO testes showed significant enrichment in biological functions related to immune and inflammatory responses (Figure 2F,G). Quantitative assessment of inflammation‐related factors further confirmed significantly elevated expression of pro‐inflammatory cytokines in testes from *Grtp1*‐KO mice, with MIP‐1g, IL‐1α, and MCSF showing marked upregulation compared to young controls (Figure S3E). To determine whether *Grtp1* deficiency recapitulates the broader transcriptional landscape of testicular aging, we performed an integrated analysis of transcriptomic data from young, aged, and *Grtp1*‐KO groups. The DEGs were classified into four functional clusters (Figure S3D). Cluster 3 (highly expressed in aged/*Grtp1*‐KO groups) primarily consisted of immune‐inflammatory‐related genes, while Cluster 4 (low in aged/*Grtp1*‐KO groups) was significantly enriched in genes associated with spermatocyte meiosis and round spermatid differentiation (Figure 2H and Figure S3G). This suggests that *Grtp1* deficiency may impair testicular function by mimicking the transcriptional regulatory network of natural aging.

Given the early‐stage impact of aging on undifferentiated spermatogonia (Uspg), we isolated THY1^+^ Uspg via fluorescence‐activated cell sorting (FACS) for RNA‐seq (Figure 2I). Dynamic expression clustering identified five gene clusters across three groups (young, aged, and *Grtp1*‐KO) (Cluster 1–5) (Figure S3H). Cluster 1 (upregulated in aged/*Grtp1*‐KO) was linked to cell cycle, apoptosis, and DNA damage response, while Cluster 2 (downregulated in aged/*Grtp1*‐KO) involved glutathione metabolism and growth factor signaling (Figure 2K,L). γH2AX staining confirmed significant DNA damage accumulation in GFRA1^+^ Uspg from both aged and *Grtp1*‐KO groups (Figure 2J). This phenotypic manifestation aligns with the observed dysregulation of genes involved in DNA damage response and related pathways within this specific cell population. Concurrently, the proportion of GFRA1^+^ cells was significantly reduced in both groups compared to the young controls (Figure S3F). These multi‐layered findings indicate that *Grtp1* deficiency likely impairs the homeostatic maintenance and regenerative capacity of this critical stem cell population by mimicking aging‐related mechanisms.

### 2.3 *Grtp1* Deficiency Drives Senescence‐Like Phenotypes in Spermatogonia via Oxidative Damage and Mitochondrial Dysfunction

**FIGURE 3:**
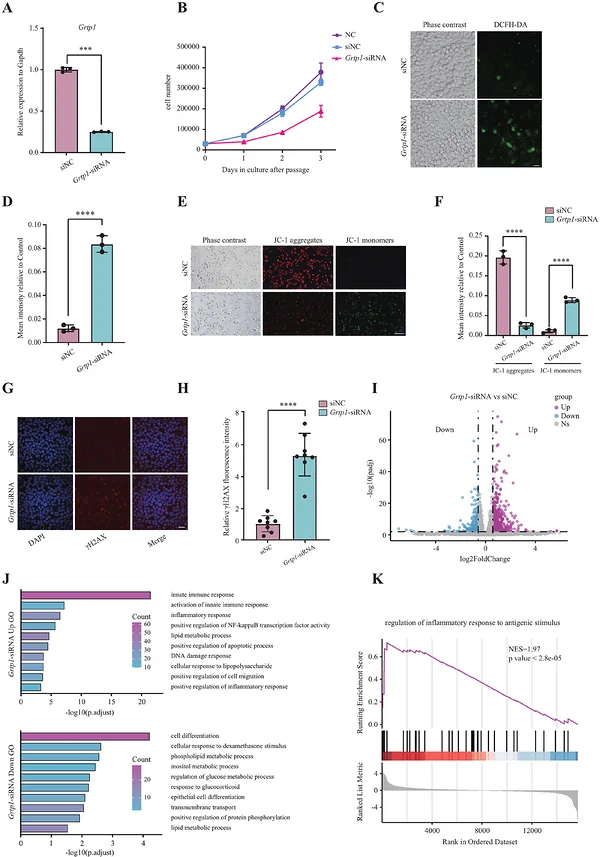
*Grtp1* deficiency drives cellular senescence in spermatogonia via oxidative stress, mitochondrial dysfunction, and genomic instability. (A) RT‐qPCR analysis of *Grtp1* mRNA levels in C18‐4 cells transfected with *Grtp1*‐siRNA vs. a scrambled siRNA control (siNC). (B) Proliferative capacity of C18‑4 cells transfected with *Grtp1*‑siRNA compared to controls (untreated NC and siNC groups). (C, D) Representative images (C) and quantification (D) of intracellular ROS levels measured by DCFH‑DA staining in C18‑4 cells transfected with *Grtp1*‑siRNA vesus siNC (scale = 100µm). (E, F) Representative images (E) and quantification (F) of mitochondrial membrane potential measured by JC‑1 staining in C18‑4 cells transfected with *Grtp1*‑siRNA vesus siNC (scale = 500µm). (G, H) Representative images (G) and quantification (H) of DNA damage assessed by γH2AX immunofluorescence staining in C18‐4 cells transfected with *Grtp1*‐siRNA vesus siNC (scale = 100µm). (I) Volcano plot of DEGs between *Grtp1*‐siRNA‐ and siNC‐transfected C18‐4 cells. (J) Significantly enriched GO biological process terms for upregulated (upper) and downregulated (lower) DEGs from the comparison in Figure 3I. (K) GSEA enrichment plot for the gene set “regulation of inflammatory response to antigenic stimulus” in *Grtp1*‐siRNA‐treated vs. siNC‐treated C18‐4 cells (NES = 1.97,  *P* < 2.8 × 10^−^^5^). Data in Figure 3A,D,F,H are shown as mean ± s.d. (*n* = 3 for a, d, f; *n* = 8 for h, from independent experiments). Significance was assessed by two‑tailed unpaired *t*‑tests (^***^ *P* < 0.001, ^****^ *P* < 0.0001).

To confirm a causal role for *Grtp1* loss in spermatogonial senescence, we employed the C18‐4 mouse spermatogonial cell line [29], a well‐established model for undifferentiated spermatogonia. Specific silencing of *Grtp1* using siRNA achieved a 75% reduction in mRNA levels (Figure 3A), and significantly impaired cellular proliferation compared to both negative (NC) and non‐targeting siRNA (siNC) controls (Figure 3B), demonstrating that *Grtp1* deficiency directly drives the spermatogonial population depletion observed in vivo.

Given the oxidative damage in *Grtp1*‐deficient testes, we hypothesized that *Grtp1* loss triggers cell‐autonomous oxidative stress and mitochondrial dysfunction. Systematic analysis in C18‐4 cells revealed *Grtp1* knockdown significantly elevated intracellular ROS levels (Figure 3C,D) and induced pronounced mitochondrial depolarization (Figure 3E,F). This redox and mitochondrial dysfunction culminated in genomic instability, as γH2AX staining revealed a pronounced increase in DNA damage foci following *Grtp1* silencing (Figure 3G,H). These results recapitulate the age‐associated cascade from oxidative stress to genomic damage.

To elucidate the transcriptomic underpinnings of *Grtp1* deficiency‐induced senescence, we performed transcriptome sequencing on siNC and *Grtp1*‐siRNA C18‐4 cells (Figure 3I). GO analysis of upregulated DEGs revealed significant enrichment in innate immune response, NF‐κB signaling, lipid metabolism, apoptosis, and DNA damage response. Conversely, downregulated DEGs were significantly enriched in biological processes related to cell differentiation and metabolism, specifically the inositol metabolic process and the regulation of glucose metabolic process (Figure 3J). This transcriptomic profile closely mirrors the patterns observed in physiological aging. Gene Set Enrichment Analysis (GSEA) further demonstrated that *Grtp1*‐KO significantly activated the “regulation of inflammatory response to antigenic stimulus” pathway (NES = 1.97, *p* < 2.8 × 10^−^^5^), indicating a role in promoting aberrant inflammation via enhanced antigenic signaling (Figure 3K). In summary, *Grtp1* deficiency establishes a pro‐senescent state in spermatogonial cells characterized by coexisting systemic inflammation and genomic instability.

### 2.4 *Grtp1* Orchestrates Redox Homeostasis via PRDX1/4‐TXN Interactions and Acetylcarnitine Metabolism

**FIGURE 4:**
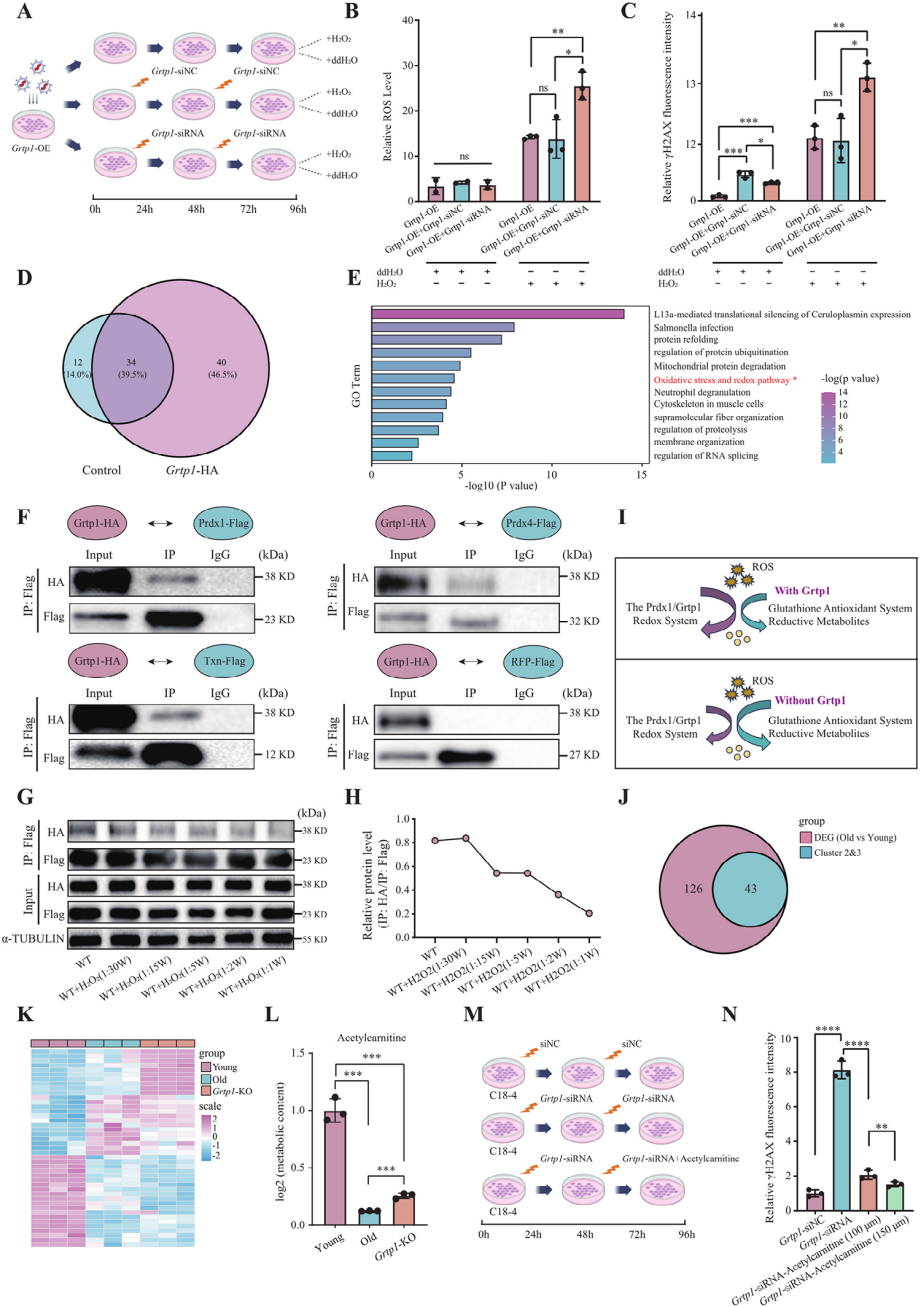
*Grtp1* maintains redox homeostasis and its deficiency‐induced oxidative damage is rescued by acetylcarnitine. (A) Schematic of the experimental design. After establishing *Grtp1* overexpression in NIH3T3 cells, cells were divided into three groups: (1) *Grtp1*‐OE (overexpression alone); (2) *Grtp1*‐OE followed by *Grtp1* knockdown (*Grtp1*‐OE + Grtp1‐siRNA); (3) *Grtp1*‐OE followed by scrambled siRNA control (*Grtp1*‐OE + siNC). (B,C) Influence of *Grtp*1 expression on cellular sensitivity to H_2_O_2_‐induced oxidative stress. B, Intracellular ROS levels measured by DCFH‐DA fluorescence. C, DNA damage assessed by immunofluorescence staining of the DNA damage marker γH2AX. (D) Venn diagram comparing proteins captured by co‐immunoprecipitation/mass spectrometry (Co‐IP/MS) from NIH3T3 cells expressing GRTP1‐HA vs. empty‐vector controls. (E) GO enrichment analysis of the 40 GRTP1‐interacting proteins, displaying the top significantly enriched biological pathways. (F) Western blot of co‑immunoprecipitation assays. Shown are HA and Flag signals in input lysates and anti‑Flag immunoprecipitates from cells co‑expressing GRTP1‑HA with PRDX1‑Flag, PRDX4‑Flag, TXN‑Flag, or an empty RFP vector control. (G) Analysis of GRTP1‐PRDX1 interaction under H_2_O_2_ treatment. Lysates from co‐expressing 293T cells exposed to an H_2_O_2_ gradient were immunoprecipitated with anti‐Flag beads and immunoblotted for HA and Flag. (H) Quantification of the GRTP1‐PRDX1 interaction. The line graph shows the HA/Flag grayscale ratio in co‐immunoprecipitates across an H_2_O_2_ concentration gradient. (I) Proposed model of the antioxidant defense network. The PRDX1/GRTP1 axis functions alongside other systems (e.g., glutathione) and reductive metabolites to form an integrated defense system. (J) Venn diagram identifying 43 metabolites common to both aged and *Grtp1*‐KO groups compared with young controls. (K) Heatmap of the 43 shared metabolites from Clusters 2 and 3, displaying their expression patterns in young, aged, and *Grtp1*‐KO groups. (L) Measured levels of acetylcarnitine in undifferentiated spermatogonia from young, aged, and *Grtp1*‐KO mice. (M) Experimental timeline for acetylcarnitine treatment in *Grtp1*‐knockdown C18‐4 cells, illustrating the administration schedule. (N) Quantification of γH2AX foci number in *Grtp1*‑siRNA C18‑4 cells after treatment with indicated concentrations of acetylcarnitine. Data in Figure 4b (n ≥ 2 per group), Figure 4C,L,N (each *n* = 3 per group) are presented as mean ± s.d. from independent experiments. Statistical significance was evaluated using two‐tailed unpaired *t*‐tests with P values indicated as ns, not significant; ^*^ *P* < 0.05, ^**^ *P* < 0.01, ^***^ *P* < 0.001 and ^****^ *P* < 0.0001.

To further investigate the role of *Grtp1* in antioxidant defense, we overexpressed *Grtp1* in NIH3T3 cells, followed by transfection with *Grtp1*‐siRNA or non‐targeting siNC (siNC). The efficacy of *Grtp1* expression modulation was confirmed by RT‐qPCR (Figure S4A). Cells were then treated with H_2_O_2_ to induce oxidative stress, and the other served as an untreated control (Figure 4A). Subsequently, intracellular reactive oxygen species (ROS) levels and γH2AX (DNA damage marker) expression were measured to assess *Grtp1*´s impact on oxidative stress resistance. As expected, H_2_O_2_ treatment induced a marked increase in both intracellular ROS levels and γH2AX across all groups, but *Grtp1* expression critically modulated damage severity. Specifically, the *Grtp1*‐siRNA group exhibited the highest ROS and γH2AX levels, while *Grtp1*‐overexpressing (*Grtp1*‐OE) group showed significant protection against both oxidative and DNA damage (Figure 4B,C and Figure S4B,C).

To delineate the molecular mechanism by which GRTP1 mitigates oxidative stress, we mapped the GRTP1 protein‐protein interaction (PPI) network in NIH3T3 cells expressing GRTP1‐HA‐P2A‐GFP, via co‐immunoprecipitation (Co‐IP) coupled with mass spectrometry. This approach identified 40 high‐confidence GRTP1‐specific interactors (Figure 4D), and Gene Ontology (GO) enrichment analysis showed significant enrichment of these proteins in oxidative stress and redox regulation pathways (Figure 4E). We validated key redox‐regulatory candidates (PRDX1, PRDX4, and TXN) via targeted Co‐IP assays. Co‐IP with anti‐Flag magnetic beads, followed by anti‐HA Western blotting, detected HA signals in both input and immunoprecipitated (IP) fractions of experimental groups, confirming GRTP1 binding to each target. No HA signals were observed in IP fractions of negative controls (Figure 4F), definitively validating these redox‐specific interactions.

To elucidate the specific role of *Grtp1* in the antioxidant process, 293T cells were co‐transfected with PRDX1‐Flag and GRTP1‐HA plasmids, then exposed to gradient H_2_O_2_ concentrations. Flag/HA signal intensities were normalized to internal controls to eliminate loading/transfection confounders. Co‐IP assays showed strong GRTP1 binding to reduced monomeric PRDX1 under basal conditions; H_2_O_2_ treatment induced dose‐dependent attenuation of this interaction, accompanied by PRDX1 conversion from reduced monomers to oxidized dimers (Figure 4G,H). This suggests GRTP1 dynamically modulates PRDX1 binding interactions to facilitate its oxidation under oxidative stress, thereby enhancing ROS clearance. In contrast, *Grtp1* deficiency impairs the PRDX1 functional cycle and compromises cellular antioxidant capacity. We next examined whether GRTP1 similarly engages PRDX4, another redox partner from our PPI screen, in a redox‐dependent manner. We therefore assessed their co‐localization under graded H_2_O_2_ concentrations. Confocal microscopy revealed substantial co‐localization of GRTP1 and PRDX4 in the cytoplasm under basal conditions. Upon H_2_O_2_ treatment, this co‐localization signal diminished in a dose‐dependent manner (Figure S5A,B), consistent with the reduced interaction observed by Co‐IP. This H_2_O_2_‐induced spatial dissociation likely reflects the oxidation‐driven shift of PRDX4 from its monomeric form to dimers, which may weaken its association with GRTP1.

Beyond the PRDX1/4 and GRTP1 axis, the organism also employs independent mechanisms such as the glutathione system and reductive metabolites to collectively form a comprehensive antioxidant defense system (Figure 4I). To identify additional redox‐regulatory metabolites, we performed untargeted metabolomics on FACS‐isolated undifferentiated spermatogonia from young (3–4M), aged (16–18 M), and *Grtp1*‐KO (3–4 M) mice. Complementary positive/negative ion‐mode mass spectrometry detected 479 metabolites, with 126 showing significant differences between young and aged groups (Figure S6A,B). KEGG pathway analysis identified significant enrichment in several biologically relevant pathways, particularly oxidation of branched‐chain fatty acids, carnitine‐acylcarnitine translocase deficiency, DNA repair, mitochondrial DNA depletion syndrome and mitochondrial fatty acid oxidation disorders (Figure S6C). Clustering of these 126 differential metabolites identified 43 shared by aged and *Grtp1*‐KO groups (Clusters 2/3), showing consistent expression trends distinct from young mice (Figures 4J,K and S6D). Among top downregulated metabolites in both aged and *Grtp1*‐KO groups, acetylcarnitine was prominently reduced (Figures 4L and S6E,F), which has known to scavenge ROS, preserve mitochondrial function, and delay senescence [30, 31].

Given acetylcarnitine´s antioxidant properties, we tested its ability to rescue *Grtp1* deficiency‐induced defects in *Grtp1*‐silenced C18‐4 cells (Figure 4M). Supplementation with 100 µm or 150 µm of acetylcarnitine significantly alleviated *Grtp1* deficiency‐induced DNA damage. Quantitative analysis through γH2AX immunofluorescence staining revealed a marked reduction in γH2AX foci number in acetylcarnitine‐treated groups compared with the siRNA control group (Figure 4N and Figure S6G). Collectively, these results establish *Grtp1* as a central regulator of redox homeostasis via PRDX1/4‐TXN interactions, and demonstrate that *Grtp1* deficiency recapitulates age‐associated metabolic perturbations, providing a genetic model to dissect complex antioxidant defense networks.

### 2.5 Precise Restoration of *Grtp1* by Injection of Growth Hormone Rescue the Effects of Aging on Testes

**FIGURE 5:**
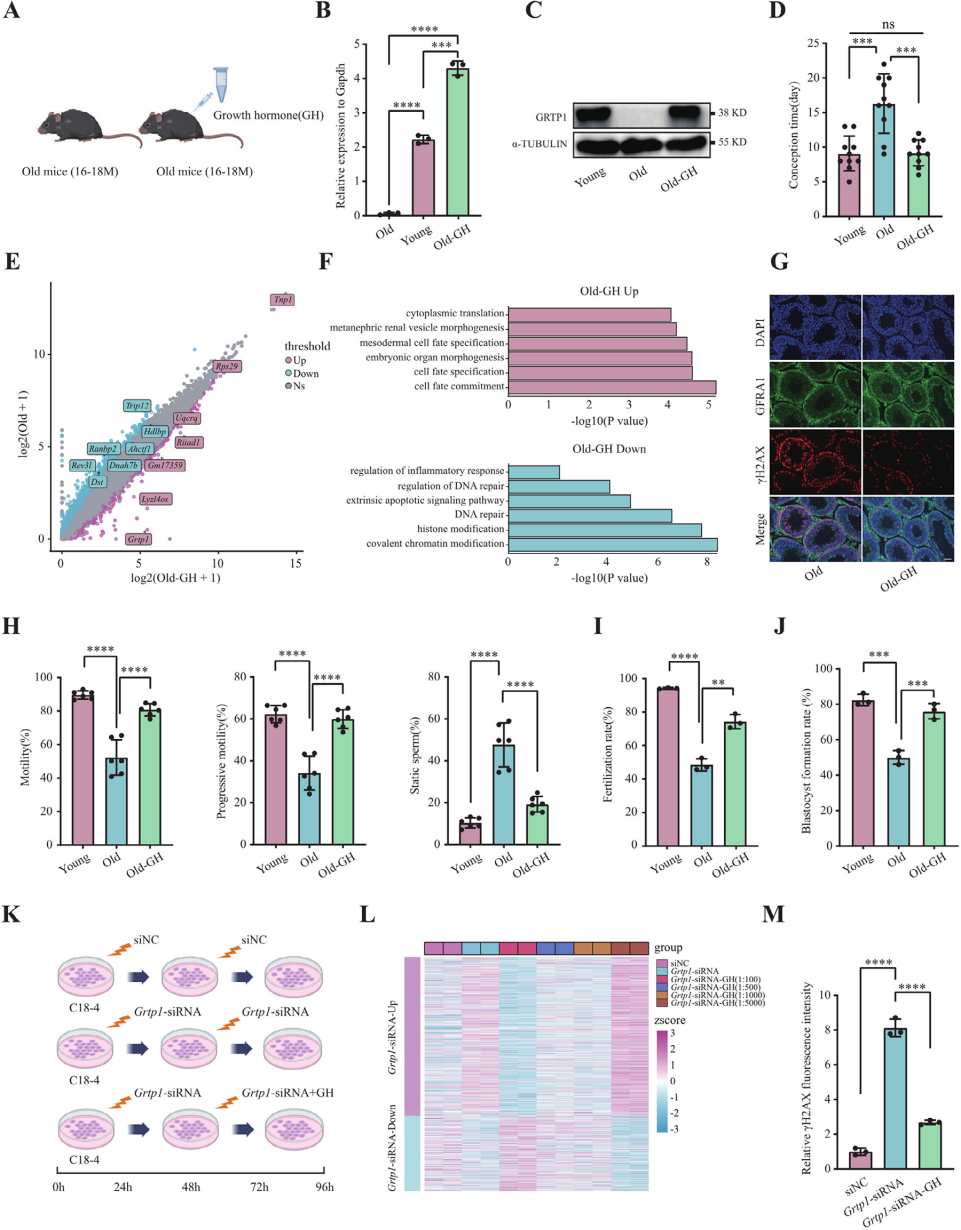
Effects of growth hormone (GH) intervention on* Grtp1* expression, testicular transcriptome, and reproductive function in aged mice. (A) Experimental design of GH intervention in aged male mice. (B) *Grtp1* mRNA expression in testicular tissues of aged mice after GH treatment, measured by RT‐qPCR. (C) Western blot analysis of GRTP1 protein levels in testicular tissues from young mice and aged mice with or without GH treatment. (D) Time from cohabitation to conception was measured for female mice paired with young males (control); or with aged male mice from either the untreated control or GH‐treated groups. (E) Volcano plot of DEGs in testicular tissues between aged and GH‐treated aged groups from mRNA‐seq. (F) GO enrichment analysis of biological processes for up‐ and down‐regulated DEGs in Old‐GH vs. Old groups. (G) Representative immunofluorescence images of testicular sections from aged control and GH‐treated mice, stained for GFRA1 (green) and γH2AX (red), with DAPI nuclear counterstain (blue) (scale = 50µm). (H) Sperm motility analysis comparing the proportions of progressively motile and static sperm in aged control and GH‐treated groups. (I) In vitro fertilization (IVF) rates using sperm from young, aged control, and GH‑treated aged mice. (J) Quantification of blastocyst development rates following in vitro fertilization (IVF) with sperm from young, aged, and GH‐treated aged mice. (K) Schematic of in vitro GH treatment in *Grtp1*‑knockdown C18‑4 spermatogonial cells. (L) Heatmap of transcriptomic changes in *Grtp1*‑knockdown C18‑4 cells treated with different GH concentrations. (M) Quantification of γH2AX fluorescence intensity in C18‑4 cells under *Grtp1* knockdown and GH treatment. Data are presented as mean ± s.d. from independent experiments, with sample sizes of *n* = 3 per group for Figure 5B, *n* = 10 per group for Figure 5D, and *n* = 6 per group for Figure 5H,I,J. Statistical significance was evaluated using two‑tailed unpaired *t*‑tests, with P values indicated as ns for not significant, ^**^ *P* < 0.01, ^***^ *P* < 0.001, and ^****^ *P* < 0.0001.

While *Grtp1* is established as a growth hormone‐responsive gene in young physiological contexts [28], it remains unknown whether GH retains this regulatory capacity in the aging organism where hormonal signaling is often dysregulated. We therefore systematically investigated GH‐induced *Grtp1* activation in an aged male mouse model (Figure 5A). A three‐week, alternate‐day GH intervention specifically activated *Grtp1* expression in aged mice (Figure 5B,C) without broadly affecting other GH target genes (Figure S7A), indicating that GH specifically activates *Grtp1* in the testis. Remarkably, female mice mated with GH‐treated aged males exhibited a significantly shorter time from cohabitation to conception compared to those mated with untreated aged males (Figure 5D), indicating that GH‐induced restoration of *Grtp1* may partially mitigate the age‐related decline in reproductive efficiency.

Based on these findings, we next sought to systematically decipher the effects of GH intervention on transcriptional regulation in aged testes. We performed mRNA‐seq analysis on testicular tissues from both aged (Old) and growth hormone‐treated aged (Old‐GH) groups, which revealed 2052 DEGs between these groups (Figure 5E). Downregulated genes showed significant enrichment in aging‐related biological processes, including regulation of inflammatory response, DNA repair, and extrinsic apoptotic signaling pathway (Figure 5F). Strikingly, these pathways precisely corresponded to the rescued phenotypes observed in our previous GH intervention experiments (such as reduced DNA damage and decreased inflammation levels), demonstrating a molecular‐level correlation between transcriptomic changes and tissue functional improvement. The immunofluorescence results demonstrated a significant reduction in H2AX phosphorylation levels in testicular tissues of aged male mice treated with growth hormone compared to untreated aged controls (Figure 5G).

We next examined whether GH intervention could improve functional outcomes in the aged reproductive system. Sperm function analysis revealed that GH treatment substantially enhanced sperm motility in aged mice: the proportion of progressively motile sperm increased by 25.8% in GH‐treated aged animals compared to untreated aged controls, accompanied by a corresponding reduction in static sperm (Figure 5H). These improvements in sperm quality translated to enhanced fertilization capacity. IVF assays revealed a 25.7% higher fertilization rate and improved early embryonic development in sperm from GH‐treated aged males compared to untreated controls (Figure 5I,J and Figure S7B).

To determine whether the restorative effects of growth hormone are mediated specifically through *Grtp1*, we first evaluated its impact on testicular tissue integrity in *Grtp1* knockout mice. γH2AX immunofluorescence analysis demonstrated that GH administration failed to reduce elevated DNA damage levels in testicular tissues of *Grtp1*‐KO mice, in contrast to the significant improvement observed in wild‐type aged mice (Figure S7C). Consistently, GH treatment did not restore sperm motility or fertilization rates in *Grtp1*‐KO mice (Figure S7D,E). These data establish *Grtp1* as an obligate mediator of GH‐induced improvements in testicular health and reproductive function.

To investigate whether GH can directly act on germ cells independently of systemic physiological complexity, we conducted GH intervention experiments in *Grtp1*‐silenced C18‐4 cells (Figure 5K). First, we confirmed in wild‐type C18‐4 cells that GH treatment upregulates *Grtp1* expression in a dose‐dependent manner (Figure S7F). Comparative transcriptomic analysis of siNC and *Grtp1*‐siRNA groups revealed that GH treatment concentration‐dependently reversed the gene expression dysregulation caused by *Grtp1* knockdown: within the concentration range of 1:100 to 1:1000, GH effectively restored normal expression patterns of differentially expressed genes; however, this restorative effect completely disappeared when the concentration was further increased to 1:5000 (Figure 5L). γH2AX immunofluorescence further confirmed GH markedly alleviated DNA damage in *Grtp1*‐knockdown C18‐4 cells (Figure 5M). These in vitro data demonstrate GH directly regulates spermatogonial gene expression and DNA integrity via *Grtp1*‐dependent mechanisms.

### 2.6 GH May Mitigate Paternal Aging and *Grtp1* Deficiency‐Induced Offspring Anxiety and Social Deficits via Correcting Sperm DNA Methylation Modifications

**FIGURE 6:**
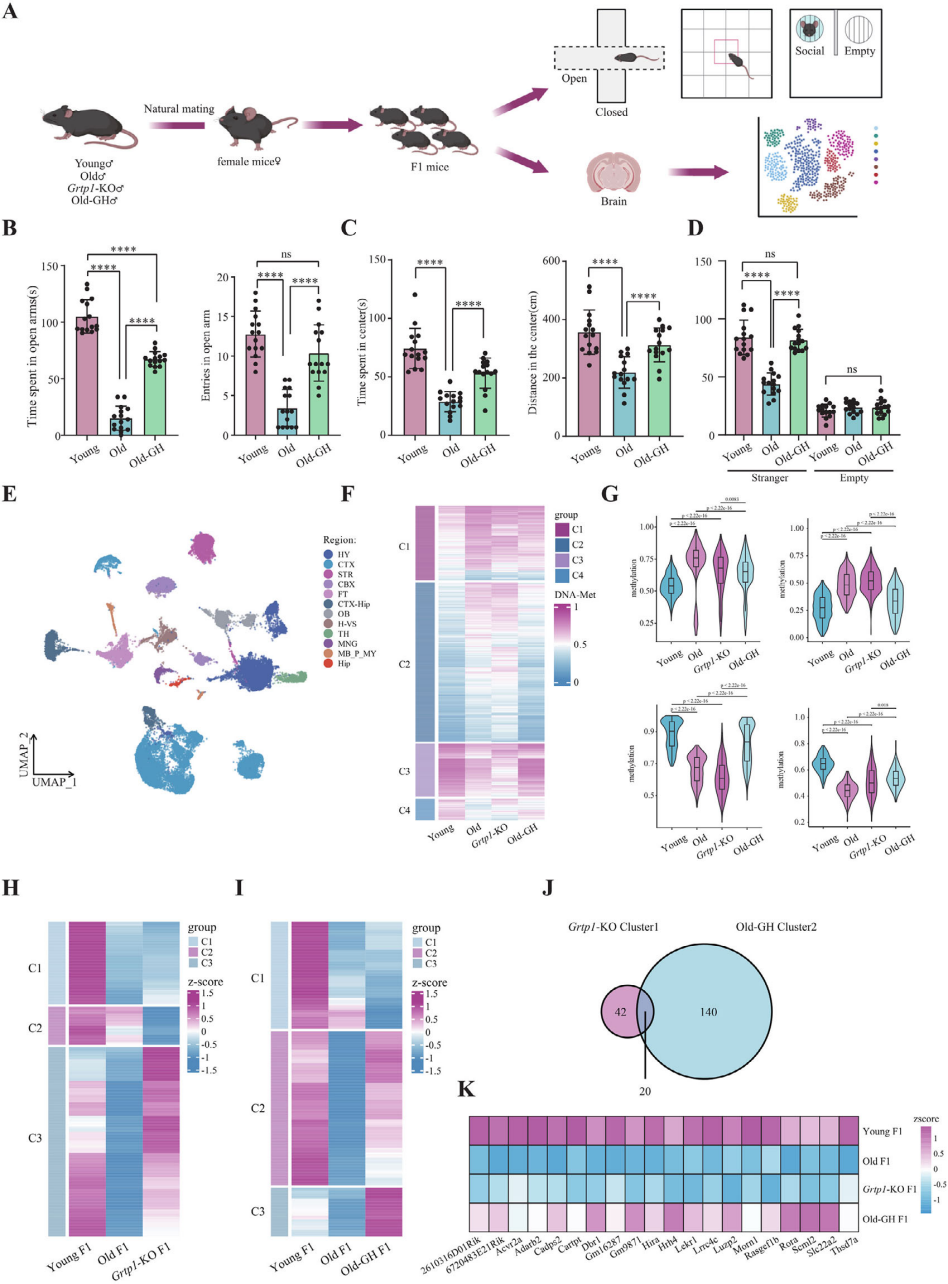
Growth hormone (GH) treatment may mitigate paternal aging‐ and *Grtp1* deficiency‐induced offspring behavioral deficits via remodeling sperm DNA methylation and restoring neurodevelopmental gene expression. (A) Schematic diagram of the experimental design. Parental male mice were divided into groups (Young, Old, *Grtp1*‐KO, Old‐GH) and naturally mated with female mice to produce F1 male offspring, which were then used for subsequent behavioral and molecular assays. (B‐D) Statistical graphs from three behavioral assays, comparing the performance of male offspring sired by Young, Old, and Old‐GH. Specifically, panel (B) shows the time spent in open arms in the elevated plus maze test; panel (C) shows the distance traveled in the center zone in the open field test; and panel (D) shows the social behavior‐related metrics in the three‐chamber social interaction test. (E) Clustering analysis of single‐cell RNA sequencing data from brain tissues. The brain cells were collected from offspring of four paternal groups (Young, Old, *Grtp1*‐KO, and Old‐GH), with each group containing brain tissues pooled from two offspring mice as one biological replicate. All cells were integrated and subjected to unsupervised clustering, resulting in multiple subpopulations annotated with brain regions, including hypothalamus (HY), cerebral cortex (CTX), striatum (STR), cerebellar cortex (CBX), fiber tracts (FT), cortical‐hippocampal region (CTX‐Hip), olfactory bulb (OB), hindbrain‐ventral spinal cord (H‐VS), thalamus (TH), meninges (MNG), midbrain, pons, and medulla (MB_P_MY), hippocampus (Hip). (F) Heatmap showing DNA methylation levels (0‐1) at young‐vs.‐aged sperm DMRs across four paternal groups (Young, Old, *Grtp1*‐KO, Old‐GH). These DMRs were classified into four clusters (C1‐C4). (G) Violin plots showing the distribution of methylation levels for each paternal group across the four clusters. (H, I) Heatmaps showing the expression of genes associated with the Cluster 2 DMR regions (defined in Figure 6G) in the hippocampus and cerebral cortex of offspring. (H) shows gene expression in offspring sired by Young, Aged, and *Grtp1*‐KO males. (I) shows gene expression in offspring sired by Young, Aged, and Old‐GH males. (J‐K) Venn diagram (J) illustrates the overlap between Cluster 1‐associated genes in the *Grtp1*‐KO paternal group and Cluster 2‐associated genes in the Old‐GH paternal group. Heatmap showing the expression of these overlapping genes in the hippocampus and cerebral cortex of offspring sired by the four paternal groups (Young, Old, *Grtp1*‐KO, and Old‐GH) (K). Data in Figure 6B–D (mean ± s.d.; *n* = 15) were analyzed by two‑tailed Student's unpaired *t*‑tests. Significance is reported as ns (not significant), ^*^ *P* < 0.05, ^**^ *P* < 0.01, ^***^ *P* < 0.001, and ^****^ *P* < 0.0001. Figure 6G was analyzed with the Wilcoxon rank‐sum test.

Leveraging evidence that advanced paternal age impairs offspring neurodevelopment [32], we used *Grtp1*‐knockout (*Grtp1*‐KO) and growth hormone (GH)‐treated aged male mouse models to systematically investigate the regulatory role of the *Grtp1* gene in this process and assess behavioral outcomes in their offspring (Figure 6A). We first performed a panel of behavioral assays on offspring sired by young, aged, and *Grtp1*‐KO male mice. In the elevated plus maze test, offspring of young males exhibited significantly longer open‐arm residence time and more open‐arm entries relative to those from aged and *Grtp1*‐KO fathers (Figure S8A). In the open field test, young paternal offspring also showed greater total locomotor activity and longer central zone exploration distance compared with the other two groups (Figure S8B). Moreover, the three‐chamber social interaction assay demonstrated that young group offspring displayed enhanced social preference, spending significantly more time interacting with stranger mice (Figure S8C). Collectively, these results demonstrate that both paternal aging and paternal *Grtp1* deficiency significantly increase the risk of anxiety‐like behaviors and social deficits in male offspring.

Notably, GH treatment significantly ameliorated behavioral impairments in offspring sired by aged male mice across all three behavioral paradigms (Figure 6B–D). Intriguingly, this beneficial effect was absent in offspring from GH‐treated aged *Grtp1*‐KO mice, indicating that the rescue of behavioral deficits relies on *Grtp1* function. Together, these data demonstrate that GH supplementation mitigates paternal aging‐induced anxiety‐like and social deficits in offspring, but exerts such protective effects in a *Grtp1*‐dependent manner.

To explore the molecular mechanisms underlying offspring anxiety and social deficits, we performed single‐cell RNA sequencing on brain tissues from four offspring groups sired by young, aged, *Grtp1*‐KO, and GH‐treated aged (Old‐GH) males, taking into account the high heterogeneity of brain tissue. Unsupervised cell clustering resolved all brain cells into 12 distinct subpopulations with well‐defined anatomical identities (Figure 6E and Figure S8D) [33]. Given that paternal aging is known to primarily affect hippocampal and cortical functions in offspring [25, 34], we used the high resolution of single‐cell profiling to specifically enrich and isolate cell populations from these two regions, laying a foundation for targeted mechanistic analyses.

To gain deeper insights into the transcriptional alterations in the brains of offspring, we performed integrative analysis of single‐cell RNA‐seq data focusing on hippocampal and cortical cell populations. Hierarchical clustering of gene expression profiles across the four offspring groups revealed a distinct gene cluster (Cluster 1) that was downregulated in the Old group and showed a similar trend in the *Grtp1*‐KO group, whereas its expression was restored to near‐young levels upon GH treatment in the Old‐GH group (Figure S9A,B). GO enrichment analysis of this cluster showed significant enrichment in neurodevelopmental and behavioral processes, including learning and memory, locomotory behavior, and neuron projection guidance (Figure S9C). This expression pattern raised the possibility that DNA methylation and other upstream epigenetic mechanisms may regulate these genes.

To explore this possibility, we analyzed whole‐genome bisulfite sequencing data for sperm from the four paternal groups. All datasets passed strict quality control thresholds (Figure S8E,F and Table S6). Comparative analysis between sperm from young and aged males identified 2256 differentially methylated regions (DMRs; ≥15% methylation difference, ≥7.5× CpG coverage), including 1630 hypermethylated and 626 hypomethylated DMRs (Figure S8G). We then performed clustering analysis of methylation levels at DMRs identified between sperm from young and aged males across the four groups of sires (Young, Old, *Grtp1*‐KO, and Old‐GH), which grouped these DMRs into four clusters (Figure 6F and Table S5). Strikingly, sperm from *Grtp1*‐KO males displayed a methylation pattern nearly identical to that of aged mice, whereas GH treatment in aged males markedly restored sperm DNA methylation toward a youthful state (Figure 6G).

To determine whether GRTP1 deficiency is sufficient to drive aberrant DNA hypermethylation independently of the complex testicular microenvironment, we knocked down *Grtp1* in C18‐4 spermatogonial cells and performed genome‐wide DNA methylation analysis. Compared with control cells, *Grtp1* knockdown resulted in 1226 DMRs (Figure S9D,E). Overlap analysis of DMR‐associated genes between in vitro Grtp1 knockdown and in vivo aging revealed 72 shared genes associated with hypermethylated DMRs, which were significantly enriched in neurodevelopmental pathways (Figure S9F,G), whereas only 42 shared genes were associated with hypomethylated DMRs, with enrichment in basic cellular processes (Figure S9H,I). These results suggest that the hypermethylation changes induced by *Grtp1* deficiency in vitro recapitulate key features of the aging‐associated sperm methylome, particularly in genes relevant to neurodevelopment. This is consistent with previous reports that sperm DNA hypermethylation in aged males is linked to neurodevelopmental pathways [35, 36]. We therefore focused on Cluster 2 DMR‐associated genes and examined their expression in the hippocampus and cerebral cortex. Compared with the young group, Cluster 1 genes were significantly downregulated in both aged and *Grtp1*‐KO offspring (Figure 6H and Figure S8H). In contrast, GH treatment specifically upregulated Cluster 2 genes in the hippocampus and cortex of offspring from aged males (Figure 6I and Figure S8I).

By overlapping genes from these two clusters, we identified 20 genes that are differentially regulated by the GH‐GRTP1 axis (Figure 6J,K). Annotation of these DMRs revealed that the majority were located in intronic and intergenic regions (Figure S8J). Among them, *Acvr2a* encodes an activin receptor in the TGFβ pathway that modulates neurodevelopment and synaptic plasticity, and its dysregulation has been suggested to be potentially associated with autism and cognitive impairment [37, 38]. *Rora*, an established high‐risk autism gene, is essential for cerebellar development, and its reduced expression correlates with structural brain abnormalities and motor deficits in ASD patients [39]. Restoring the expression of these key neurodevelopmental genes likely represents a core mechanism through which GH supplementation alleviates paternal aging‐associated neurobehavioral deficits in offspring. Thus, GH may mitigate paternal aging and *Grtp1* deficiency‐induced offspring behavioral deficits via sperm DNA methylation remodeling and subsequent restoration of neurodevelopmental gene expression.

## 3 Discussion

The accumulation of oxidative damage and exacerbation of DNA damage have been widely recognized as hallmark alterations in the testicular tissue of aging male mice [40]. However, the key upstream regulatory factors that mediate the occurrence of these damages or directly govern the testicular aging process remain largely unidentified. To address this, we performed integrated multi‐omics profiling and functional genetic analysis at single‐cell resolution. Our scRNA‐seq data revealed that transcriptional dysregulation first arises in the foundational population of undifferentiated spermatogonia, and through a strategic cross‐sex transcriptome comparison, we pinpointed the testis‐enriched factor *Grtp1* as a male‐specific and aging‐sensitive candidate. The comprehensive recapitulation of the aged testicular phenotype‐including elevated oxidative stress, mitochondrial dysfunction, DNA damage in spermatogonia, systemic inflammation, and severe impairment of sperm function‐in young *Grtp1*‐KO mice provides compelling causal evidence that *Grtp1* deficiency is a driver, rather than a consequence, of reproductive aging. Importantly, its age‐related downregulation positions the GH‐GRTP1 signaling axis as a potential therapeutic target. Furthermore, our finding that paternal *Grtp1* deficiency is sufficient to induce neurobehavioral deficits in offspring offers a valuable genetic model for future investigations into the paternal origins of anxiety‐like and social behavioral deficits.

At the mechanistic level, a key breakthrough of this study is the elucidation of GRTP1's novel function as a dynamic redox sensor within spermatogonia. We found that GRTP1 forms a functional interaction module with core antioxidant proteins, PRDX1/4 and TXN. It is well‐established that the PRDXs (including PRDX4) and TXN system constitute a core network for cellular defense against oxidative damage [41, 42]. Our proposed “stress‐and‐release” model further reveals that GRTP1 actively optimizes ROS clearance efficiency by facilitating the transition of PRDX1 from reduced monomers to oxidized, active dimers and its subsequent dissociation, thereby establishing GRTP1 as a key regulatory node within this canonical antioxidant defense network. Having established GRTP1's antioxidant role, we sought to position it within the broader redox network. The observation that GRTP1‐mediated protection is contingent on the integrity of the PRDX/TXN system (Figure S5C,D) suggests that GRTP1 functions in concert with the PRDX/TXN system, rather than through an independent parallel pathway. This epistatic relationship implies that the thiol‐reducing capacity provided by PRDX/TXN is an indispensable downstream effector arm for the GH signal.

If GRTP1 functions as a central regulatory hub, then its disruption would be expected to unmask latent compensatory circuits that maintain redox balance under stress. Our metabolomic data further demonstrate that the loss of this central regulatory node triggers a compensatory cellular response, characterized by a marked depletion of acetylcarnitine, a critical metabolite known for scavenging free radicals and supporting mitochondrial function. The fact that exogenous supplementation of acetylcarnitine could partially rescue the DNA damage phenotype not only validates its protective role but also reveals the existence of a metabolic compensation mechanism‐a backup antioxidant strategy mobilized when the GRTP1‐mediated core pathway is compromised. Nevertheless, the precise mode of action underlying acetylcarnitine's protective effect remains to be defined. Acetylcarnitine is well‐recognized both as a direct ROS scavenger and as a carrier for mitochondrial fatty acid oxidation that supports bioenergetic homeostasis; therefore, its beneficial actions could be mediated through direct neutralization of oxidative stress, through improvement of mitochondrial energy metabolism, or through indirect cross‐talk with the PRDX1/4‐TXN antioxidant axis. While our current data do not distinguish among these possibilities, it is plausible that these mechanisms operate in concert, given the interconnected nature of redox balance and mitochondrial function. Future studies employing pathway‐specific inhibitors or genetic manipulations will be required to dissect the relative contributions of each mechanism to the observed phenotypic rescue. Ultimately, the age‐related functional decline of this integrated system, which combines precise regulatory control with metabolic responses, leads to the accumulation of oxidative damage at the molecular, organellar, and genomic levels.

It should be noted that the detailed dissection of the GRTP1‐PRDX1 molecular interaction and its stress‐responsive dynamics in this study was performed in heterologous cell systems (NIH/3T3 and HEK293T). This approach was necessitated by the practical challenges of conducting sophisticated protein biochemistry in primary spermatogonia. These cells served as a clear and controllable platform for probing fundamental protein‐protein interactions. The successful reconstitution of this functional module in non‐germ cells demonstrates that the redox‐regulatory capability is an intrinsic property of the GRTP1 protein itself. This finding reinforces our central thesis that the severe phenotypic consequences of *Grtp1* loss in the testis stem from the unique physiological reliance of male germ cells on this finely‐tuned regulatory node to cope with their exceptional metabolic and oxidative demands, whereas other cell types may possess compensatory pathways.

At the translational level, the most encouraging finding is the efficacy of GH in reversing the aging phenotype. We demonstrated that GH treatment specifically reactivates *Grtp1* expression in aged individuals, thereby ameliorating the testicular redox state, reducing DNA damage and inflammation, and ultimately significantly improving sperm quality and fertilization rates. Critically, the complete absence of any therapeutic benefit from GH in *Grtp1*‐KO models unequivocally establishes GRTP1 as the indispensable downstream effector of GH action. Nevertheless, the precise signaling cascade through which GH upregulates *Grtp1* transcription remains to be defined. Moreover, because our in vivo GH administration was systemic, the observed upregulation of *Grtp1* in testicular tissue could result from direct GH action on germ cells, indirect effects mediated through somatic cells, or both, a distinction that our C18‐4 spermatogonial cell data alone cannot fully resolve. In addition, the applications of GH has been controversial [43, 44]. Furthermore, whether GH regulates *Grtp1* expression in testicular somatic cell types such as Sertoli cells, Leydig cells, or peritubular myoid cells remains to be determined. Future studies using conditional knockout or cell type‐specific GH receptor deletion will be necessary to systematically dissect the direct germ cell effects from indirect somatic cell‐mediated effects.

Beyond the testis, our work elucidates a clear pathway from the paternal germline to the offspring's brain. The nearly identical anxiety‐like behaviors and social deficits in offspring derived from either aged or *Grtp1*‐KO fathers strongly argue that paternal GRTP1 deficiency is a key mechanism transmitting the risk for anxiety‐like and social behavioral deficits. The reversal of these behavioral abnormalities and the restoration of neurodevelopmental gene expression in the brains of offspring from GH‐treated aged fathers‐an effect strictly dependent on GRTP1‐provides a definitive interventional link for this intergenerational phenomenon. Nevertheless, it is important to recognize that behavioral abnormalities in offspring of aged males likely arise from multifactorial mechanisms, and *Grtp1* deficiency represents one of several contributory factors rather than the sole determinant.

Beyond oxidative DNA damage, our multi‐omic analyses suggest that sperm DNA methylation remodeling may represent an additional layer of intergenerational information transfer. Comparative WGBS profiling revealed that *Grtp1* deficiency recapitulates the age‐associated sperm methylome landscape, while GH treatment in aged males substantially restores a youthful methylation pattern. Integrated analysis of sperm DMRs and offspring brain scRNA‐seq data identified 20 candidate genes, including the neurodevelopmental regulators *Acvr2a* and *Rora*, whose expression changes correlated with paternal methylation status. Although these correlative data do not establish causality, they raise the intriguing possibility that GH‐*Grtp1* signaling safeguards offspring neurobehavioral outcomes not only by limiting oxidative DNA damage but also by preserving proper sperm DNA methylation patterns. Nonetheless, it must be acknowledged that DNA methylation changes likely contribute only partially to the overall neurobehavioral phenotype. Given the complexity of intergenerational inheritance, other epigenetic layers (e.g., histone modifications, small non‐coding RNAs) and environmental factors may also play substantial roles in shaping offspring behavioral outcomes.

Several limitations of this study should be acknowledged, as they point to fruitful directions for future research. First, while we focused on the GRTP1‐PRDX1 axis, the functional significance of GRTP1's interactions with other redox regulators like TXN remains to be fully elucidated. Second, the precise molecular cargo in sperm responsible for transmitting the behavioral deficits to offspring awaits identification. Our observation that *Grtp1* deficiency leads to significantly increased genomic instability in sperm prompts us to propose a hypothesis: the oxidative DNA damage accumulated in paternal germ cells due to GRTP1 loss may be transmitted to the zygote through some yet‐undefined mechanism (for instance, resulting in sperm carrying certain specific DNA damage or mutations), thereby interfering with early embryonic development, particularly the programming of the nervous system, and ultimately manifesting as neurobehavioral abnormalities in the offspring. Validating this hypothesis will be a key focus of future research. Furthermore, the systemic administration of GH used in this study means that its benefits likely result from a combination of specific *Grtp1* reactivation and other body‐wide improvements. Finally, our behavioral assessment was primarily focused on anxiety‐like and social behaviors, and did not encompass other domains commonly associated with neurodevelopmental disorders, such as repetitive/stereotypic behaviors or cognitive flexibility. Thus, the current behavioral battery may not capture the full spectrum of neurobehavioral consequences resulting from paternal aging and *Grtp1* deficiency, and future studies incorporating a more comprehensive behavioral test battery will be valuable to delineate the broader neurodevelopmental impact.

In conclusion, we propose a unified model: age‐related decline in testicular GH signaling leads to reduced GRTP1 expression, thereby disrupting the redox regulatory network orchestrated by GRTP1 and its partners, including PRDX1. Given the unique dependency of male germ cells on this network, its failure triggers oxidative damage, genomic instability, and inflammation in spermatogonia, ultimately impairing spermatogenesis and altering the molecular composition of sperm. These paternal “scars” manifest concurrently as reduced fertility and an increased risk of neurobehavioral deficits in offspring. Our work identifies GRTP1 as a critical contributor to the maintenance of paternal germline integrity and suggests that the GH‐GRTP1 axis may represent a promising target for developing strategies to mitigate the reproductive and intergenerational health challenges associated with advanced paternal age. However, given the multifactorial etiology of offspring neurobehavioral outcomes and the correlative nature of some of our intergenerational data, further studies are required to fully establish the causal chain and to evaluate the translational potential of targeting this axis in clinical settings.

## 4 Experimental Section

### 4.1 Animal Procedures

We used C57BL/6 male mice as study subjects, with young and old groups defined as 3–4 months (3–4 M) and 16–18 months (16–18 M) of age, respectively, based on established age thresholds in murine aging studies. The *Grtp1* knockout mice were generated and housed at the Animal Experiment Center of Tongji University. All experimental procedures and animal breeding protocols were conducted in accordance with the ethical guidelines for the use of experimental animals at Tongji University.

### 4.2 The Design and Validation of *Grtp1* Knockout

The CRISPR/Cas9 components, including Cas9 mRNA and sgRNAs, were prepared as previously described [45]. Briefly, both components were generated by in vitro transcription, and paired sgRNAs targeting the second exon of the *Grtp1* gene were designed based on the C‐CRISPR principle. The two sgRNAs targeting the *Grtp1* gene were: *Grtp1*‐sgRNA1 (5’‐TCACCAAGAGGGCGATCAAATGG‐3’) and *Grtp1*‐sgRNA2 (5’‐TAAAAGGAAACGGTGGTGTCCGG‐3’). For genotyping, PCR was performed using *Grtp1*‐F1 (5’‐TGTCGGGTGTCGGAGCTCTTG‐3’) and *Grtp1*‐R1 (5’‐CCTGCAGTCCAGAGGTCTCG‐3’).

### 4.3 Digestion of Testicular Tissue and Preparation of Single‐Cell Suspension

Testes were collected from young and aged male mice (n = 2 per group) following cervical dislocation. The tissues were decapsulated, and the seminiferous tubules were washed 2–3 times with DPBS. Subsequently, the tubules were minced on ice with scissors and digested with 2 mg/mL collagenase IV for 15 min. For any remaining tissue fragments, further dissociation was carried out using 0.05% trypsin. Finally, the final suspension was washed, and one sequencing library was constructed for each group using the 10X Genomics platform.

### 4.4 Flow Cytometric Sorting of Spermatogonial Subtypes

Undifferentiated and differentiated spermatogonia were isolated by flow cytometry using antibodies against THY1 (BioLegend, #140310) and C‐KIT (BioLegend, #135108), respectively, as previously described [46].

### 4.5 Cell Culture

The mouse undifferentiated spermatogonial cell line C18‐4 was obtained from Qingqi (Shanghai) Biotechnology Development Co., Ltd., and maintained in DMEM/F‐12 medium (Thermo Fisher Scientific, 11320082) supplemented with 10% fetal bovine serum (Sigma–Aldrich, F7524), 1× GlutaMAX (Fisher Scientific, 35‐050‐061), 1× non‐essential amino acids (Fisher Scientific, 11‐140‐050), and 1× penicillin‐streptomycin. NIH3T3 and HEK‐293 cells were cultured in DMEM (Life Technologies, C11960500BT) containing 10% qualified fetal bovine serum of Australian origin (Thermo Fisher Scientific, 10099141) and 1× GlutaMAX.

### 4.6 siRNA Transfection

The siRNA targeting the *Grtp1* gene and the negative control (siNC) were synthesized by Shanghai Genema. For PRDX/TXN pathway disruption, siRNAs targeting *Prdx4* or *Txn* were designed and synthesized accordingly. Transfection was performed using the VigoFect transfection reagent according to the manufacturer's protocol. Briefly, cells were seeded 24 h prior to transfection, reaching 60%–70% confluence at the time of transfection. The siRNA and VigoFect reagent were separately diluted with sterile saline, mixed in equal volumes, and incubated at room temperature for 15 min to form transfection complexes. The complexes were then added dropwise to the cell culture medium. After 6–8 h, the medium was replaced with fresh complete medium. Cells were harvested 48 h post‐transfection, and the knockdown efficiency of *Grtp1* was evaluated by RT‐qPCR. All siRNA sequences are provided in Table S1.

### 4.7 Untargeted Metabolomic Profiling and Analysis

Untargeted metabolomics was performed on undifferentiated spermatogonia isolated from the testes of young, aged, and *Grtp1*‐KO mice. Metabolites were extracted from cell pellets and then separated and analyzed by UPLC‐QToF‐MS (ACQUITY UPLC BEH amide column; AB Sciex TripleTOF 6600) in both positive and negative ESI modes. Quality control samples were integrated to ensure reliability. Metabolites were identified by matching accurate mass and MS/MS spectra (m/z tolerance < 25 ppm) to a standard database [47]. Orthogonal Partial Least Squares Discriminant Analysis (OPLS‐DA) was performed using the R software to visualize the overall metabolic separation between groups. For the definitive identification of differential metabolites, a univariate analysis was applied based on the criteria of abs(log2FC) > 0.5 and a padj < 0.05. Metabolome enrichment analysis was conducted using MetaboAnalyst 6.0. The untargeted metabolomics profiling was conducted by Applied Protein Technology Co., Ltd. (Shanghai, China). The relative levels of all detected untargeted metabolites are provided in Table S2.

### 4.8 Immunofluorescence Staining

The testicular tissues were fixed overnight at 4°C with 4% paraformaldehyde and then subjected to frozen sectioning. The sections were permeabilized with 0.5% Triton X‐100 for 30 min at room temperature, followed by blocking with 3% BSA‐PBS for 1–2 h at room temperature. The sections were then incubated overnight at 4°C with a mixture of γH2AX antibody (1:500, Cell Signaling Technology, #9718) and GFRA1 antibody (1:500, Santa Cruz Biotechnology, sc‐271546). After washing three times with 0.5% BSA‐PBS, the sections were incubated for 1 h at room temperature in the dark with a mixture of corresponding anti‐mouse and anti‐rabbit fluorescent secondary antibodies. Finally, nuclei were stained with DAPI(Invitrogen, D3571) for 15–20 min at room temperature. For immunofluorescence staining of γH2AX in cells, the cells were first fixed with 4% paraformaldehyde and then blocked with 3% BSA‐PBS for 1 h at room temperature. The samples were subsequently incubated overnight at 4°C with γH2AX primary antibody (1:500, Cell Signaling Technology, #9718), washed three times with 0.5% BSA‐PBS, and incubated for 1 h at room temperature with an anti‐rabbit secondary antibody. Nuclei were counterstained with DAPI using the same protocol as described above.

### 4.9 Sperm Motility Assessment

Sperm motility was assessed using a computer‐assisted sperm analysis (CASA) system. Briefly, sperm samples were collected from the cauda epididymis and allowed to capacitate in a 35°C water bath for 10–15 min. A 10 µL aliquot was then placed on a pre‐warmed chamber slide for computer‐assisted analysis.

### 4.10 Assessment of Sperm Function by In Vitro Fertilization

Sperm were collected from the epididymis and vas deferens of male mice euthanized by cervical dislocation. The cauda epididymis was punctured with a needle to release sperm into G‐IVF medium (Vitrolife, Sweden), followed by a 20–30 min incubation. Motile sperm from the upper layer were then collected and co‐cultured with cumulus‐oocyte complexes for in vitro fertilization. After fertilization, the zygotes were washed and transferred to G1‐PLUS medium (Vitrolife, #10128) for subsequent culture.

### 4.11 Quantitative Analysis of GRTP1‐PRDX1 Interaction Intensity

To investigate the dynamic interaction between GRTP1 and PRDX1 under oxidative stress, HEK293T cells were co‐transfected with plasmids encoding GRTP1‐HA and PRDX1‐Flag. At 48 h post‐transfection, the cells were treated with a gradient concentration of H_2_O_2_ for 1h to induce varying degrees of oxidative stress. Following treatment, the cells were lysed, and the clarified lysates were subjected to immunoprecipitation using Anti‐Flag Magnetic Beads (Selleck Chemicals, #B26101) to capture PRDX1‐Flag and its associated proteins. The immunoprecipitated complexes were analyzed by Western blotting. The membranes were probed with an anti‐HA antibody (Cell Signaling Technology, #3724) to detect co‐precipitated GRTP1‐HA and an anti‐Flag antibody (Smart‐Lifesciences, #SLAB0102) to determine the total levels of immunoprecipitated PRDX1‐Flag. The interaction intensity between GRTP1 and PRDX1 under different oxidative stress conditions was quantitatively assessed by calculating the signal intensity ratio of GRTP1‐HA to PRDX1‐Flag (HA/Flag).

### 4.12 In Vivo Growth Hormone Administration

Recombinant mouse growth hormone (mGH; BioVision, #4770‐1000) was reconstituted in sterile deionized water to a concentration of 0.1–1.0 mg/mL. Aged male mice (16–18 months old) received intraperitoneal injections of the mGH solution at a dosage of 10 µg per 10 g of body weight. The injections were administered every 48 h for a total duration of three weeks. This regimen was demonstrated to activate the expression of the *Grtp1* gene.

### 4.13 Mouse Behavioral Assays

Behavioral tests were conducted at the Animal Experiment Center of Tongji University, using the Labmaze V3.0 system for automated tracking and recording. The experimental subjects were F1 offspring (10–12 weeks old, n = 15 per group) derived from crossing five groups of male mice (Young, Aged, *Grtp1*‐KO, Old‐GH, *Grtp1*‐KO+GH) with C57BL/6J female mice. All mice were acclimatized for one week prior to testing, and all apparatuses were cleaned with 75% ethanol between trials to eliminate olfactory cues. The specific testing procedures were as follows: (1) Elevated Plus Maze Test: The number of entries into and the time spent in the open and closed arms were recorded during a 5‐min session to assess anxiety‐like behavior (less time spent in the open arms indicates higher anxiety). (2) Open Field Test: The time spent in and the number of entries into the central area of the arena were recorded over 5 min to evaluate anxiety levels based on thigmotaxis. (3) Three‐Chamber Social Interaction Test: During the 10‐min test, the time spent by the mouse in the chamber containing an unfamiliar mouse (stranger) vs. the chamber with an empty wire cup was recorded to analyze social interaction preference.

### 4.14 Gene Ontology Analysis

To identify the functional categories of differentially expressed genes, GO analysis of biological processes was performed using the enrichGO function from the R package clusterProfiler v3.14.3 [48] using the org.Mm.eg.db database. Dot plots were generated using the dotplot function, which is part of clusterProfiler.

### 4.15 Single‐Cell RNA‐seq Data Processing

We processed two batches of single‐cell RNA‐seq data from distinct tissues: testis and brain. The testis dataset was generated using the 10X Genomics platform, while the brain dataset was prepared using DNBelab C4 technology. The analytical workflows for each dataset are described separately below.

Testis Data Processing: Sequencing reads from the testis samples were aligned to the mouse genome (mm9) using CellRanger v2.0.2 (10X Genomics). Subsequent analyses were performed using Maestro, a comprehensive Snakemake‐based pipeline that integrates several dozen bioinformatics tools and packages [49]. This workflow facilitated the complete processing sequence from alignment and sample integration to quality control, cell filtering, normalization, and unsupervised clustering. We identified cell types based on the expression of known marker genes. Specifically, we merged count matrices from both young and aged samples, selecting cells with over 500 detected genes and less than 5% mitochondrial gene expression. We also identified 2000 highly variable genes for dimensionality reduction, retaining 40 principal components. To identify differentially expressed genes (DEGs) between cell clusters in testicular tissues of young vs. aged male mice, we applied thresholds of an average log_2_ fold change (avg_log2FC) > 0.5 and an adjusted p‐value (padj) < 0.05. The resulting gene lists are provided in Table S3.

Brain Data Processing: The brain dataset was initially processed with DNBC4tools. Subsequent analyses were conducted using Seurat, where we performed quality control, filtering, routine preprocessing, and unsupervised clustering on the expression matrix. Additionally, Gene Set Variation Analysis (GSVA) was utilized to score cells based on highly expressed genes from various brain regions reported in the brain spatial transcriptome literature [50]. This scoring assisted in annotating the cells. Similar to the testis data, we filtered cells with over 500 detected genes and mitochondrial gene expression below 5%, selected 2000 high variability genes for dimension reduction, and retained 50 principal components. These tailored workflows enabled rigorous, reproducible analyses of single‐cell transcriptomic data, capturing cellular heterogeneities and providing insights into the cellular composition of the testis and brain under different conditions. DEGs were identified in two comparisons within the hippocampus and cerebral cortex of male offspring: (1) young vs. aged groups, and (2) aged control vs. aged with growth hormone injection. In both analyses, a stringent threshold of an average log_2_ fold change (avg_log2FC) > 1 and an adjusted *p*‐value (padj) < 0.05 was applied to define significance.

### 4.16 RNA‐seq Library Preparation for Mouse Testis Tissue

Total RNA was isolated from mouse testis tissues using TRIzol reagent. RNA quality was verified by gel electrophoresis and quantified using a Qubit system. Sequencing libraries were prepared from 500 to 1000 ng of total RNA using the KAPA Stranded RNA‐Seq Library Preparation Kit according to the manufacturer's protocol. The procedure involved RNA fragmentation, first‐ and second‐strand cDNA synthesis, end repair and A‐tailing, adapter ligation, and PCR amplification. Final libraries were purified with Ampure XP Beads and subjected to 150‐bp paired‐end sequencing on an Illumina Nova6000 platform.

### 4.17 Bulk RNA‐seq Data Processing

Adapters were removed from RNA‐seq data, and low‐quality reads were trimmed using Trim_galore (version 0.6.4) with the default parameters. The processed reads were then mapped to the mm9 reference genome by STAR (version 2.7.1a) [51] with the parameters –dta‐cufflinks –no‐mixed –no‐discordant. The expression levels for all Refseq genes were quantified to FPKM by Cufflinks (version 2.2.1) [52], and FPKM values of replicates were averaged for each gene. For genes with multiple isoforms, the longest transcripts were selected. The read counts for each gene were calculated using FeatureCounts (version 2.0.0) [53], and DESeq2 (version 1.26.0) [54] were used for gene differential expression analysis. DEGs were identified using the following thresholds: for the Young vs. Old and *Grtp1*‐siRNA vs. siNC comparisons, abs(log_2_FC) > 0.58 and adjusted *p*‐value (padj) < 0.05; for the *Grtp1*‐KO vs. Young and Old‐GH vs. Old (Aged) control comparisons, abs(log_2_FC) > 1 and padj < 0.05. All DEG lists are provided in Table S4.

### 4.18 WGBS

For WGBS, genomic DNA was extracted from sperm and C18‐4 cells, with 50 ng per replicate and two biological replicates per group. To monitor bisulfite conversion efficiency, Lambda DNA was added into each sample prior to bisulfite treatment. Bisulfite conversion was performed using the MECOV50 Bisulfite Conversion Kit (Scientific) according to the manufacturer's protocol. The converted DNA was then subjected to library preparation using the EpiArt DNA Methylation Library Kit for Illumina V3 (NE103, Vazyme), with 8 cycles of PCR amplification. Paired‐end 150‐bp sequencing was performed on a HiSeq X10 platform (Illumina).

### 4.19 Identify Differentially Methylated Regions (DMRs)

We applied the methpipe software (v1.3.0) [55] to generate the differentially methylated regions (DMRs) between old and young samples. In brief, we first calculated the differential methylation score of each CpG site using the methdiff program. Next, we calculated the hypomethylated regions using the hmr program and used the dmr program to identify DMRs. A DMR was defined as a region spanning at least 5 CpGs and having a dmsr (dmsr = number of differentially methylated CpGs/number of CpGs) greater than 0.15. The functional enrichment for genes that are near DMRs was analyzed using the GREAT tool with default settings [56].

### 4.20 Western Blot

Total proteins were extracted from testicular tissues or relevant cells using RIPA lysis buffer containing protease and phosphatase inhibitors. After quantification by BCA assay, proteins were denatured, separated by SDS‐PAGE, and transferred to PVDF membranes. The membranes were blocked, then incubated with primary antibodies (rabbit polyclonal anti‐GRTP1, Absin, abs148639; 1:200; rabbit monoclonal anti‐HA Tag, Cell Signaling Technology, #9718; 1:500; mouse monoclonal anti‐Flag Tag, Smart‐Lifesciences, SLAB0102; 1:500; mouse monoclonal anti‐Alpha Tubulin, Proteintech, 66031‐1‐Ig; 1:2000) at 4°C overnight, followed by incubation with HRP‐conjugated secondary antibodies at room temperature for 1h. Protein signals were detected by chemiluminescence.

### 4.21 RT‐qPCR

Various organ tissues, including testicles, were placed in 1 mL TRIzol reagent (Invitrogen) and 3–4 RNase‐free grinding beads in a homogenizer to extract RNA. cDNA was synthesized using 5X All‐In‐One RT Master Mix. Quantitative PCR (QPCR) was performed using Chamq Universal SYBR qPCR Master Mix (Vazyme, Q711‐02, China). The signals were detected using a real‐time PCR system (ABI7500, Applied Biosystems, USA). Gapdh was used as an endogenous control. The qPCR primers for *Grtp1* were forward, 5’‐TGGAGACGGTGCTACGGAT‐3’ and reverse, 5’‐ GGTCGTCATGGACAGGCTTC‐3’. The results were analyzed with GraphPad Prism 8.0 software.

### 4.22 Quantification and Statistical Analysis

Statistical analyses were performed using GraphPad Prism software. Detailed statistical information for the data presented in this study, including the applied statistical tests, the value and definition of the sample size n, and the degree of data dispersion and accuracy indicators, are described in the figure legends.

## Author Contributions

W.Q.L., Y.G., and S.R.G. conceived and designed the experiments. Y.D.L. and S.Y.L. performed the experiments. H.X.L. and Y.P.Z. performed the computational analysis. J.N.Y., Y.H.L., J.Q.Y., X.G.Z., Y.Z., D.D.B., Z.H.Y., Z.A.Z., Y.P.J., K.S.L., Y.F.S., J.N.X., C.X.X., B.X.D., X.Y.L., J.Y.C., H.W. assisted with the experiments. Y.D.L., W.Q.L., and S.R.G. wrote the manuscript.

## Conflicts of Interest

The authors declare no conflicts of interest.

## Supporting information




**Supporting File 1**: advs77463‐sup‐0001‐SuppMat.docx.

**Supporting File 2**: advs77463‐sup‐0002‐TableS1.xlsx.

**Supporting File 3**: advs77463‐sup‐0003‐TableS2.xlsx.

**Supporting File 4**: advs77463‐sup‐0004‐TableS3.xlsx.

**Supporting File 5**: advs77463‐sup‐0005‐TableS4.xlsx.

**Supporting File 6**: advs77463‐sup‐0006‐TableS5.xlsx.

**Supporting File 7**: advs77463‐sup‐0007‐TableS6.xlsx.

## Data Availability

The datasets used in this study are available from the Genome Sequence Archive (GSA) under accession numbers CRA012082, CRA012117, CRA012112, CRA041163, and CRA047973. CRA012082 contains single‐cell RNA‐seq data from the testis of young and aged male mice, as well as from the brain of F1 offspring derived from young, aged, *Grtp1*‐KO, and growth hormone‐injected aged male mice, also includes bulk mRNA‐seq data from *Grtp1*‐knockdown C18‐4 cells and cells treated with various concentrations of growth hormone. CRA012117 comprises bulk mRNA‐seq data from testicular tissues of young, aged, *Grtp1*‐KO, and growth hormone‐injected aged male mice. CRA012112 provides bulk mRNA‐seq data from undifferentiated (THY1^+^) and differentiated (C‐KIT^+^) spermatogonia isolated from young, aged, and *Grtp1*‐KO mice. Furthermore, the WGBS‐seq dataset CRA041163 was obtained from sperm of *Grtp1*‐KO and growth hormone‐injected aged (Old‐GH) male mice, and dataset CRA047973 was generated from *Grtp1*‐knockdown C18‐4 cells and control cells.
