# Supplementary material for: Grtp1 Safeguards Against Paternal Reproductive Aging and Intergenerational Behavioral Deficits by Preserving Redox Homeostasis
Source: Adv Sci (Weinh). 2026 Sep 27:e77463. Online ahead of print. doi: 10.1002/advs.77463 (PMC13616408; doi:10.1002/advs.77463)
Supplement: Supplementary file 1 [file ADVS-9999-e77463-s003.docx]

**Supplemental Data**

***Grtp1* safeguards against paternal reproductive aging and intergenerational behavioral deficits by preserving redox homeostasis**

Yingdong Liu^1#^ | Shanyao Liu^1#^ | Haixin Liang^1#^ | Jiani Yang^1^ | Yanhe Li^1^ | Jiqing Yin^1^ | Xueguang Zhang^1^ | Yan Zhang^1^ | Dandan Bai^1^ | Zihui Yan^1^ | Zhuoao Zhang^1^ | Yanping Jia^1^ | Kuisheng Liu^1^ | Yifan Sheng^1^ | Jiani Xiang^1^ | Chenxiang Xi^1^ | Baoxing Dong^1^ | Xinyi Lei^1^ | Jiayu Chen^1^ |Hong Wang^1^ | Yi Guo^1^* | Yanping Zhang^1^* | Shaorong Gao^1^* | Wenqiang Liu^1^*

**Supplementary Tables**

**Table S1.** Sequences of the siRNAs used in this study, including targeting *Grtp1*, *Prdx4*, *Txn*, and the non-targeting negative control (siNC).

**Table S2.** Abundance levels of untargeted metabolites detected in undifferentiated spermatogonia from the testes of young, aged, and *Grtp1*-KO male mice.

**Table S3.** Lists of differentially expressed genes (DEGs) for each cell cluster derived from the single-cell RNA sequencing analysis, comparing testes from young versus aged male mice.

**Table S4.** Full lists of differentially expressed genes (DEGs) identified in the following comparisons: Young vs. Old, *Grtp1*-siRNA vs. siNC, *Grtp1*-KO vs. Young, and Aged-GH vs. Aged control.

**Table S5.** DNA methylation levels at differentially methylated regions (DMRs) identified between sperm from young and aged males, shown across four paternal groups (Young, Old, *Grtp1*-KO, and Old-GH).

**Table S6.** Quality control statistics of WGBS-seq data for sperm from *Grtp1*-KO and Old-GH male mice.

**Figure S1 | Histological, inflammatory factor, and transcriptomic validation associated with male reproductive aging.** (A) Representative H&E-stained testicular sections from young and aged mice. Aged testes exhibit degenerative alterations, including seminiferous tubule atrophy and thinning of the seminiferous epithelium. (B) Analysis of inflammatory mediators in young and aged testicular tissues via quantitative protein array. Data show significant upregulation of the pro-inflammatory factors MIP-1g (**p* < 0.05), IL-1a (***p* < 0.01), and ICAM-1 (**p* < 0.05) in aged tissues. MCSF showed an increasing trend (p = 0.072) but did not reach statistical significance. (C) UMAP feature plots depicting the expression of representative marker genes used for annotating the testicular cell types presented in Fig. 1C. (D) Bar plot displaying the number of differentially expressed genes (DEGs) between young and aged groups for each testicular cell type. (E) Volcano plot derived from bulk mRNA-seq analysis comparing testicular tissues of young and aged mice. (F) Gene Ontology (GO) enrichment analysis of up- and down-regulated DEGs identified from bulk mRNA-seq of aged versus young mouse testicular tissues. Figure S1B were analyzed by two‑tailed Student’s unpaired t‑tests. Significance is reported as ns (not significant), **P* < 0.05, ***P* < 0.01.

**Figure S2 | Screening and identification of *Grtp1* as a key downregulated factor in male reproductive aging through multi-dimensional comparative transcriptomics.** (A) Screening strategy for candidate aging-associated genes. The Venn diagram illustrates the intersection between two gene sets: (left) genes highly expressed in young male testes compared to young female ovaries, and (right) genes upregulated in young versus aged male testes, resulting in seven overlapping candidate genes. (B) Heatmap showing the expression of the seven candidate genes in young testes, aged testes, and young ovaries. (C) Volcano plots showing significant differentially expressed genes (adjusted p-value < 0.05, abs(log₂FC) > 0.5) that are representative of transcriptional changes between young and aged mice across distinct testicular cell types.

**Figure S3 | Validation and functional analysis of the *Grtp1*-KO model.** (A) Sanger sequencing chromatograms confirming the specific 29‑bp deletion in exon 2 of *Grtp1*‑KO mice compared to young controls. (B) Representative bright‑field images of pre‑implantation embryos at the 2‑cell and blastocyst stages derived from IVF using sperm from young, aged, and *Grtp1*‑KO mice (scale = 50 μm). (C) Line chart depicting the developmental rates of pre‑implantation embryos at successive stages following IVF in young, aged, and *Grtp1*‑KO groups. (D) Heatmap of differentially expressed genes (DEGs) from bulk testicular RNA‑seq of young, aged, and *Grtp1*‑KO mice. DEGs are grouped into four clusters based on expression pattern similarity. (E) Violin plots of pro‑inflammatory cytokine levels (MIP‑1γ, IL‑1α, MCSF) in testicular tissues from young and *Grtp1*‑KO mice, quantified by protein array. (F) Quantification of GFRA1-positive undifferentiated spermatogonia cell proportions in testicular sections from young, aged, and *Grtp1*-KO groups. (G) GO enrichment analysis of biological processes for the four gene clusters identified in Fig. 1D. (H) Expression patterns of five gene clusters (Clusters 1‑5) identified by time‑series analysis of transcriptomic data from FACS‑sorted THY1⁺ undifferentiated spermatogonia and C‑KIT⁺ differentiating spermatogonia across young, aged, and *Grtp1*‑KO groups. Data in Figure S3b are presented as mean ± s.d. (n = 2 independent experiments). In Figure S3E, data distribution is shown as violin plots from n = 3 independent experiments; statistical comparison was performed using two‑tailed unpaired Student’s t‑tests. Data in Figure S3F are presented as mean ± s.d. (n = 10 independent experiments) and were analyzed by two‑tailed unpaired Student’s t‑tests. Significance is denoted as ns (not significant), **P* < 0.05, ***P* < 0.01, *****P* < 0.0001.

**Figure S4 | *Grtp1* expression modulation and oxidative stress response in NIH3T3 cells.** (A) RT-qPCR analysis validating the efficiency of *Grtp1* overexpression and siRNA-mediated knockdown in NIH3T3 cells. (B) Representative fluorescence microscopy images of intracellular ROS levels detected by DCFH-DA staining in NIH3T3 cells under different *Grtp1* expression conditions with or without H₂O₂ treatment (scale = 500 μm). (C) Representative immunofluorescence images of γH2AX foci (indicating DNA damage) in NIH3T3 cells under different *Grtp1* expression conditions with or without H₂O₂ treatment.

**Figure S5 | GRTP1-PRDX4 co-localization and GH-dependent DNA damage protection via PRDX/TXN signaling.** (A-B) Confocal microscopy analysis of GRTP1-PRDX4 co-localization in cells co-expressing GRTP1-GFP and PRDX4-RFP under indicated concentrations of H₂O₂ (Scale = 5 μm). Quantification of co-localized fluorescence intensity is shown in (B). (C-D) Representative immunofluorescence images (C) and quantification (D) of γH2AX foci in C18-4 cells subjected to knockdown of *Prdx4* or *Txn*, followed by GH treatment with or without H₂O₂ challenge (Scale = 10 μm). Figure S5B, D were analyzed by two‑tailed Student’s unpaired t‑tests. Significance is reported as ns (not significant), ns, not significant; **P* < 0.05, *****P* < 0.0001.

**Figure S6 | Metabolomic profiling of undifferentiated spermatogonia and acetylcarnitine rescue of *Grtp1* deficiency-induced DNA damage.** (A) Principal Component Analysis (PCA) of metabolomic profiles from FACS-sorted undifferentiated spermatogonia of young, aged, and *Grtp1*-KO groups. (B) Heatmap of differentially abundant metabolites between young and aged undifferentiated spermatogonia. (C) KEGG pathway enrichment analysis of the differentially expressed metabolites between young and aged undifferentiated spermatogonia. (D) Heatmap showing the expression patterns of 126 young-versus-aged differential metabolites across young, aged, and *Grtp1*-KO groups, categorized into four distinct clusters. (E,F) Bar graphs displaying the top 10 most significantly upregulated (E) and downregulated (F) metabolites, respectively, that are common to both aged and *Grtp1*-KO groups compared to the young group. **(**G**)** Representative immunofluorescence images of γH2AX foci in *Grtp1*-knockdown C18-4 cells following treatment with the indicated concentrations of acetylcarnitine.

**Figure S7 | Effects of GH intervention in aging models and *Grtp1*-KO mice.** (A) Expression levels of classical GH-target genes in testicular tissues from young, aged, and GH-treated aged mice. (B) Representative bright-field images of preimplantation embryos at different developmental stages derived from IVF using sperm from aged mice with or without GH treatment. (C) Immunofluorescence analysis and quantification of γH2AX foci in testicular tissues from *Grtp1*-KO mice with or without GH treatment. (D) CASA of sperm motility parameters in *Grtp1*-KO mice with or without GH treatment. (E) In vitro fertilization (IVF) rates using sperm from *Grtp1*-KO mice with or without GH treatment. (F) Bar graph showing *Grtp1* mRNA expression levels in wild-type C18-4 cells treated with a gradient of GH concentrations.

**Figure S8 | Supplemental behavioral, quality control, and expression data related to GH effects on paternal aging and *Grtp1* deficiency.** (A-C) Behavioral comparisons of offspring sired by young, aged, *Grtp1*‑KO, or *Grtp1*‑KO-GH males in the elevated plus maze (A), open field test (B), and three‑chamber social test (C). (D) Expression heatmap of canonical marker genes used to identify major brain regions in the single‑nucleus RNA‑sequencing dataset. (E-F) Quality assessment of WGBS-seq data. (E) Distribution of DNA methylation levels across CpG sites. (F) Relationship between DNA methylation level and CpG density. CpG sites were classified based on the number of CpG dinucleotides within a 100 bp flanking region. CpG sites with low density exhibited higher methylation levels, whereas those with high density showed lower methylation levels. (G) Statistics of differentially methylated regions (DMRs) numbers between sperm from young and aged males. (H-I) Heatmaps showing the methylation levels of specific DMR cluster-associated regions in the hippocampus and cerebral cortex of offspring. (H) shows the methylation levels of Cluster 1 DMR regions in the Young, Aged, and *Grtp1*-KO paternal groups (corresponding to Cluster 1 in Figure 6H). (I) shows the methylation levels of Cluster 2 DMR regions in the Young, Aged, and Old-GH paternal groups (corresponding to Cluster 1 in Figure 6I). (J) Genomic distribution of the 20 DMRs associated with the candidate genes. Data in Figure S8B,D (mean ± s.d.; n = 15) were analyzed by two‑tailed Student’s unpaired t‑tests. Significance is reported as ns (not significant), **P* < 0.05, ***P* < 0.01, ****P* < 0.001, and *****P* < 0.0001.

**Figure S9 | Clustering analysis of offspring brain single-cell transcriptomes and in vitro assessment of DNA methylation changes upon *Grtp1* knockdown.** (A) Heatmap showing four gene expression clusters (C1-C4) in hippocampal and cortical cells from offspring of Young, Aged, *Grtp1*-KO, and Old-GH fathers. (B) Box plots showing the expression distribution of each cluster across the four offspring groups. (C)

GO enrichment analysis of the four gene clusters identified from hierarchical clustering across the four offspring groups (Young, Old, *Grtp1*-KO, and Old-GH). (D) Number of DMRs identified upon *Grtp1* knockdown in C18-4 cells compared with control cells. (E) Heatmap showing the methylation levels of the DMRs identified in (D). (F)  Overlap of hypermethylated DMR-associated genes between in vitro and in vivo conditions. (G) GO enrichment analysis of the 72 shared hypermethylated DMR-associated genes. (H) Overlap of hypomethylated DMR-associated genes between in vitro and in vivo conditions. (I) GO enrichment analysis of the 42 shared hypomethylated DMR-associated genes.

**Figure S1 | Histological, inflammatory factor, and transcriptomic validation associated with male reproductive aging.**

**Figure S2 | Screening and identification of *Grtp1* as a key downregulated factor in male reproductive aging through multi-dimensional comparative transcriptomics.**

**Figure S3 | Validation and functional analysis of the *Grtp1*‑KO model.**

**Figure S4 | *Grtp1* expression modulation and oxidative stress response in NIH3T3 cells.**

**Figure S5 | GRTP1-PRDX4 co-localization and GH-dependent DNA damage protection via PRDX/TXN signaling.**

**Figure S6 | Metabolomic profiling of undifferentiated spermatogonia and acetylcarnitine rescue of *Grtp1* deficiency-induced DNA damage.**

**Figure S7 | Effects of GH intervention in aging models and *Grtp1*-KO mice.**

**Figure S8 | Supplemental behavioral, quality control, and expression data related to GH effects on paternal aging and *Grtp1* deficiency.**

**Figure S9 | Clustering analysis of offspring brain single-cell transcriptomes and in vitro assessment of DNA methylation changes upon *Grtp1* knockdown.**
